# Stimuli-responsive membrane activity of cyclic-peptide–polymer conjugates†
†Electronic supplementary information (ESI) available. See DOI: 10.1039/c9sc00756c


**DOI:** 10.1039/c9sc00756c

**Published:** 2019-04-18

**Authors:** Matthias Hartlieb, Sylvain Catrouillet, Agnès Kuroki, Carlos Sanchez-Cano, Raoul Peltier, Sébastien Perrier

**Affiliations:** a Department of Chemistry , University of Warwick , Gibbet Hill Road , Coventry CV4 7AL , UK . Email: s.perrier@warwick.ac.uk; b Faculty of Pharmacy and Pharmaceutical Sciences , Monash University , 381 Royal Parade , Parkville , VIC 3052 , Australia; c Warwick Medical School , The University of Warwick , Coventry CV4 7AL , UK

## Abstract

Cyclic peptide nanotubes (CPNT) consisting of an even number of amino acids with an alternating chirality are highly interesting materials in a biomedical context due to their ability to insert themselves into cellular membranes. However, unwanted unspecific interactions between CPNT and non-targeted cell membranes are a major drawback. To solve this issue we have synthetized a series of CPNT–polymer conjugates with a cleavable covalent connection between macromolecule and peptide. As a result, the polymers form a stabilizing and shielding shell around the nanotube that can be cleaved on demand to generate membrane active CPNT from non-active conjugates. This approach enables us to control the stacking and lateral aggregation of these materials, thus leading to stimuli responsive membrane activity. Moreover, upon activation, the systems can be adjusted to form nanotubes with an increased length instead of aggregates. We were able to study the dynamics of these systems in detail and prove the concept of stimuli responsive membrane interaction using CPNT–polymer conjugates to permeabilize liposomes as well as mammalian cell membranes.

## Abstract 2

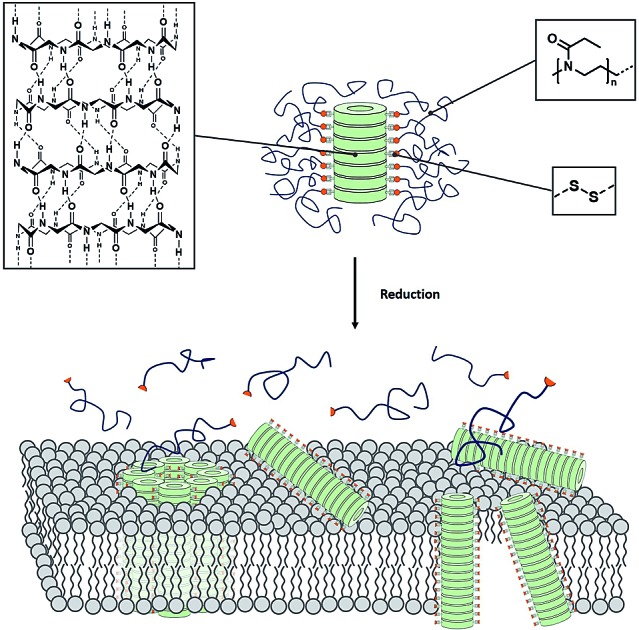
Cyclic peptide nanotubes were coupled to poly(oxazoline)s using a cleavable connection. Upon stimuli responsive detachment of the polymer an on-demand membrane activity could be achieved.

## Introduction

Hybridization of DNA and the folding of proteins are just two examples of processes based on hydrogen bonding essential for biological systems.^1^ While this interaction is used extensively in nature, the utilization of H-bonds in aqueous environment, to *i.e.* form stable supramolecular assemblies, is often challenging as a result of the competitive hydrogen bonding abilities of water. Hence, when designing supramolecular polymers for an aqueous environment, multivalent interactions or hydrophobic protection of the binding sites is usually required.^2^ Still, supramolecular polymers and in particular supramolecular biomaterials based on hydrogen bonding interactions represent a major share of the investigated systems.^3^ Examples for such materials include, but are not limited to, short β-sheet-forming peptide sequences which can self-assemble into nanofibers under the right conditions,^4^ ureido-pyrimidinone,^5^ bis-urea motifs,^6^ benzene-1,3,5-tricarboxamides,^7^ or peptide amphiphiles.^8^

Nanotubes based on cyclic peptides (CP) exhibiting an even number of amino acids with an alternating chirality are another fascinating class of supramolecular polymers.^9^ They were first synthesized by Ghadiri, Granja and coworkers in 1993 10 and soon utilized as antimicrobial materials^11^,^12^ due to their strong interaction with (cellular) membranes.^13^ Multivalent hydrogen bonding enables stacking of CP and formation of long tubular assemblies. Interestingly, the supramolecular nature of these cyclic peptide nanotubes (CPNT) enables them to readily exchange subunits and adjust to environmental conditions.^14^ This, combined with their ability to form supramolecular structures in aqueous media, makes CPNT exciting biomaterials. However, the presence of lateral aggregation between nanotubes that hampers the solubility of these systems, as well as the lack of membrane specificity were major drawbacks for the use of the original design in biomedical applications.

These issues can be overcome by the combination of CPNT with polymers by covalent conjugation.^15^,^16^ Using macromolecules of different nature and size it is possible to control the length and colloidal stability (by using *i.e.* charged polymers,^17^ or steric interactions),^15^,^18^ or to impart functionality to the supramolecular assemblies.^19^

One remarkable property of CPNT is their ability to form pores and channels in lipid bilayers including cellular membranes.^13^,^20^,^21^ While this is an intriguing feature, specificity towards selected membranes at a predetermined time point is often lacking as evidenced by high levels of haemotoxicity in antimicrobial CPNT.^11^ It has been described in prior publications that Janus conjugates are able to form macropores,^21^ and that a CPNT carrying a thermo-responsive polymer shell is able to generate pores into liposome membranes as a response to an increase in temperature.^20^

**Scheme 1:**
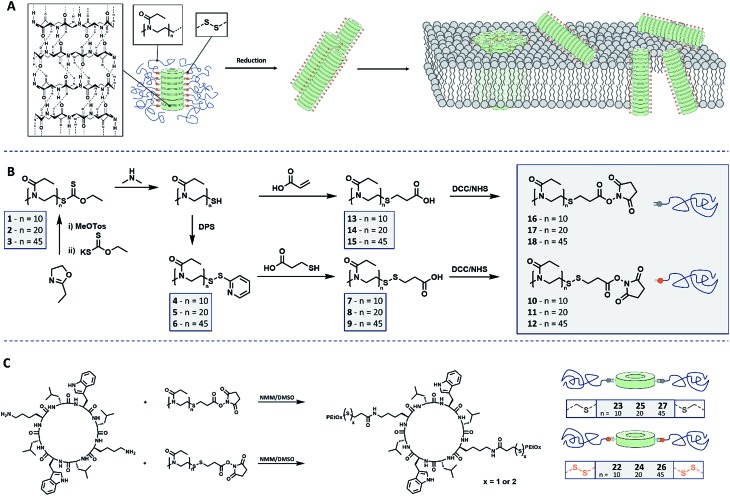
Schematic representation of (A) cyclic peptide–polymer conjugates connected by reversible covalent connections and reduction induced membrane interaction. (B) Synthetic route leading to PEtOx including cleavable and non-cleavable connectors. (C) Conjugation strategy to yield stimuli responsive CPNT conjugates.

In order to develop this approach further we envisioned a stimuli responsive activation mechanism to induce membrane activity of CPNT on demand. Therefore, a polymer is to be conjugated to the peptide in a reversible covalent fashion, and cleaved upon a change of the environmental conditions. If the polymer is chosen appropriately, it will form a shell around the CPNT that will protect the nanotube from any undesired interactions with membranes and other surfaces. Cleavage of the polymer should result in release of the CPNT and a consequent pore formation/membrane disruption (Scheme 1A).

## Results and discussion

Poly(2-ethyl-2-oxazoline) (PEtOx) was selected to constitute the shell of the materials as it is (a) hydrophilic and biocompatible^22^ and (b) easy to functionalize using a variety of different approaches.^23^ Herein we present the synthesis of CPNT–PEtOx conjugates with a cleavable disulphide linker between polymer and peptide enabling the stimuli responsive detachment of the polymeric shell. It is envisioned that upon cleavage of the linker, an on-demand membrane activity can be generated whereas initial conjugates remain inactive due to the hydrophilic polymer shell (Scheme 1A). Disulphides were chosen as their cleavage can be accurately controlled by the addition of chemical stimulus (reducing agent), while being comparably stable under ambient conditions. In addition, the environment of certain cellular compartments can lead to an activation of the compounds event tough the focus of this work is the creation of stimuli responsive CPNT and on the detailed understanding of their behavior and dynamics.

For the synthesis of the polymeric component of the conjugate, cationic ring-opening polymerization (CROP) of 2-ethyl-2-oxazoline (EtOx) was employed. In order to produce PEtOx which can be linked reversibly to a CP, ethyl xanthate was used to quench the polymerization.^24^ This precursor was then cleaved by aminolysis to yield thiol end groups,^25^ which in turn were transferred into either reduction cleavable or non-cleavable end groups, able to be connected to CP (Schemes 1B and S1†).

To generate cleavable end groups, thiols were activated using dipyridyl sulfide, which was subsequently substituted by thiopropionic acid. The carboxylic acid was activated towards amine groups by an *N*-hydroxylsuccinimide (NHS) group. The amidation reaction was chosen to connect CP and polymer as it proceeds at room temperature, in contrast to prior procedures using PEtOx.^26^ In the case of non-cleavable systems the thiol was functionalized with acrylic acid in a Michael-addition resulting in a (non-responsive) thioether connection. The acid end group was likewise activated with an NHS unit.

To evaluate the identity and quantity of the desired end groups after each functionalization step NMR spectroscopy and ESI mass spectrometry were used. In each case the degree of functionalization (DF) of the polymer chain was above 0.8 (Fig. S1–S14, Table S3†). Size exclusion chromatography (SEC) measurements proved the absence of chain coupling during the reactions. Using the described approach, it was possible to use the same parent polymer to create both, responsive and non-responsive materials, which ensures comparable properties of both polymers. To study the influence of the polymer length on the self-assembly of conjugates and more importantly, on the stimuli responsive properties of these materials, PEtOx with three different degree of polymerization (DP) values (10, 20 and 45) were produced. All initial polymers showed low dispersities that remained unaffected by the respective functionalization routes. A detailed overview over the polymer synthesis can be found in the ESI.†

**Fig. 1:**
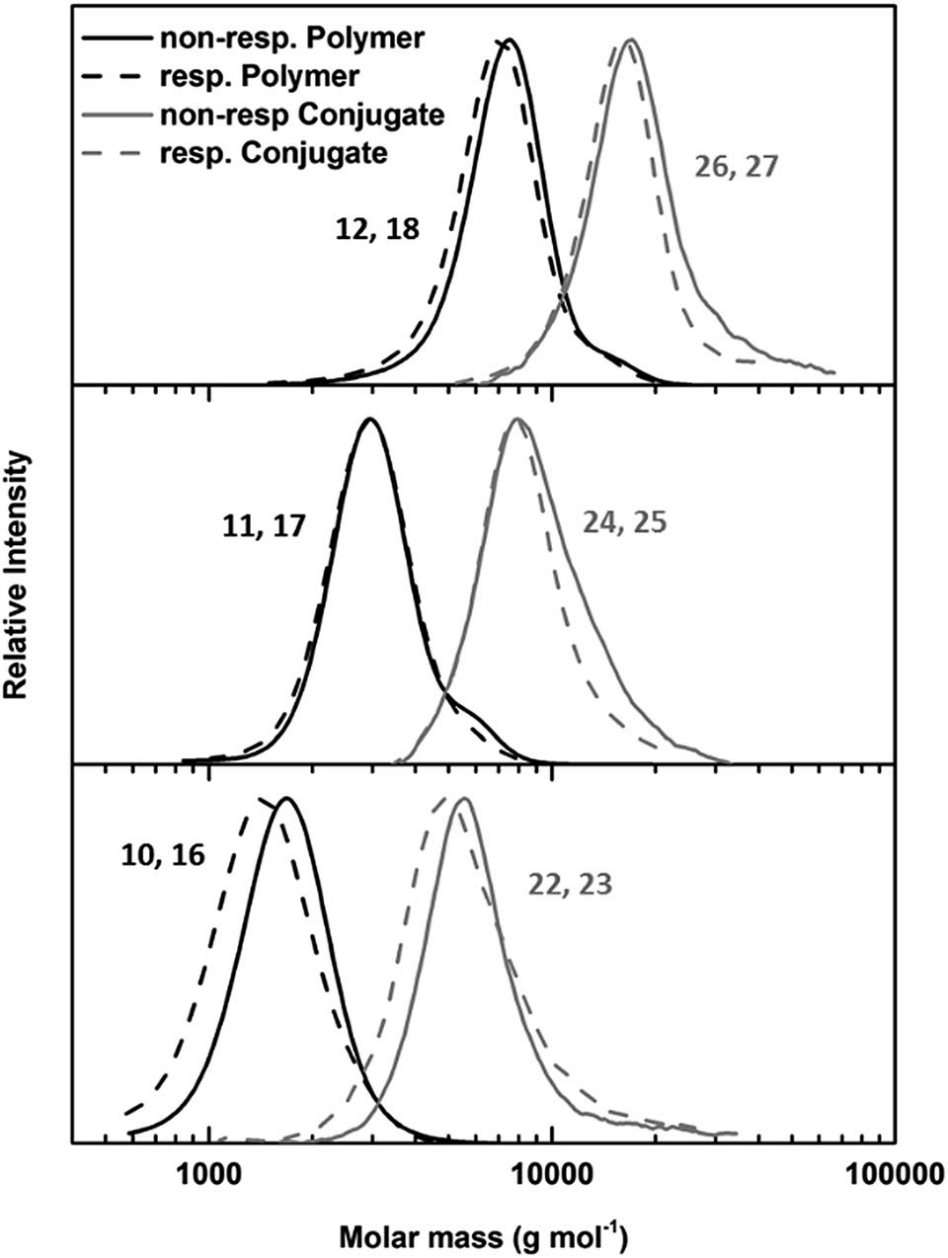
SEC of responsive (dotted lines) and non-responsive (solid lines) PEtOx polymers (black lines) and PEtOx–CP conjugates (grey lines) with DP values 45 (top), 20 (middle) and 10 (bottom).

The key component for the design of stimuli responsive nanotubes is the cyclic peptide used as building block. Cyclo(l-Trp–d-Leu–l-Lys–d-Leu–l-Trp–d-Leu–l-Lys–d-Leu) (**21**) was used, as the formation of nanotubes using this peptide in combination with a polymeric shell is well established.^17^,^20^,^27^,^28^A linear peptide precursor was synthesized in a solid phase approach using Fmoc chemistry and cleaved from the resin without deprotection. Then, cyclization was performed at low concentration connecting *N*- and *C*-terminus using DMTMM as coupling agent to limit racemization,^29^ and the subsequent deprotection led to cyclic peptides that could be conjugated to two polymeric chains. All peptides were analysed by ESI-ToF, NMR and HPLC (Scheme S2, Fig. S15–24†). Peptide **21** possesses two lysine units on opposite sides of the cycle served as anchor point for the attachment of polymers (Scheme 1C). The conjugation of polymers and peptides was performed in DMSO using a base to activate the lysine moieties. A 1.25-fold excess of polymer was used to ensure a quantitative amidation, and excess polymer was removed by centrifugation-assisted dialysis. The success of the reaction was followed by SEC in DMF/LiBr (to ensure the absence of β-sheet mediated stacking; Fig. 1). The presence of a monomodal distribution at approximately twice the molecular weight of the precursor polymer indicates quantitative conjugation as two polymer chains are connected by one cyclic peptide, and also proves the absence of unreacted polymers. Biological environments pose a challenge to structures based on hydrogen bond interactions as aqueous environment may affect the supramolecular interactions. To assess the state of self-assembly in water, static light scattering (SLS) was employed. SLS is a powerful technique used to determine the molar mass of self-assembled systems in solution and their number of aggregation, *N*_agg_ (Fig. S21–24†).

As depicted in Table 1, the number of aggregation of conjugates in water is highly dependent on the DP of the attached polymer arms. Conjugates with PEtOx_10_ arms were not water soluble, and therefore, no light or neutron scattering experiments could be conducted for these materials. However, conjugates with PetOx_45_ arms form nanotubes with a number of aggregation of around 30, corresponding to a tubular length around 15 nm. No significant difference between responsive and non-responsive systems was detected, indicating the presence of an intact polymer shell for cleavable nanotubes under ambient conditions. When the DP of the PEtOx arms was lowered to 20 a strong increase in aggregation can be observed. This is the result of a decrease in steric hindrance caused by the attached polymers, and is in accordance with prior studies.^18^ Furthermore, a pronounced difference between responsive and non-responsive tubes carrying (PEtOx)_20_ arms was observed. Whereas non-responsive nanotubes possessed a number of aggregation around 90 (42 nm), a 3.5 fold increase was detected for responsive tubes (*N*_agg_ = 278, 131 nm).

**Table 1:** Characterization data of PEtOx–CP conjugates

Sample	Precursor polymer		SEC^*c*^		SLS					SANS	
	Connection	DP	*M*_n_ (g mol^–1^)	*Đ*	*M*_a_ (g mol^–1^)	*N*_agg_	L^*b*^ (nm)	d*n*/d*c*	*R*_g_ (nm)	*L* (nm)	*N*_agg_
22	CH_2_–S–S–CH_2_	10	5 100	1.25	—^*a*^	—^*a*^	—^*a*^	—^*a*^	—^*a*^	—^*a*^	—^*a*^
23	CH_2_–S–CH_2_	10	5 600	1.33	—^*a*^	—^*a*^	—^*a*^	—^*a*^	—^*a*^	—^*a*^	—^*a*^
24	CH_2_–S–S–CH_2_	20	7 800	1.10	1 600 000	278	131	0.191	27	190	400
25	CH_2_–S–CH_2_	20	8.600	1.22	450 000	90	42	0.190	38	27	58
26	CH_2_–S–S–CH_2_	45	15 100	1.11	340 000	34	16	0.175	30	6.6	14
27	CH_2_–S–CH_2_	45	16 800	1.22	320 000	32	15	0.170	22	5.9	13

^*a*^Materials could not be analysed due to insolubility in water.

^*b*^calculated from *N*_agg_.

^*c*^Measured in DMF with 0.1 % LiBr, using a poly(methyl methacrylate) calibration.

While SLS provides information on the absolute molecular weight and number of aggregation, small angle neutron scattering (SANS) provides insights about the shape of the nano-object. As both methods operate at complementary *q* range, and signals from both methods can be transformed into an apparent molecular weight (*M*_a_, see ESI† for more information), a coherent picture can be drawn by the combination of the two methods (Fig. S25–S36†).^30^

Various structural models, including spherical shape, rod or Gaussian chains, were fitted to the data obtained from the combination of SLS and SANS experiments. However, only a cylindrical micelle model could be fitted appropriately to the scattering data. This form factor was already used to fit similar self-assembling systems and describes the expected architecture well.^17^,^28^,^31^ Furthermore, by knowing the distance between two neighboring cyclic peptides within a nanotube,^10^,^18^,^32^ it is possible to calculate the *N*_agg_ from the nanotube length obtained from the structural model fitted to the SANS data. Interestingly, this value of *N*_agg_ was similar to the value derived solely from SLS data (Table 1), although the generally lower values in SANS are best explained by the presence of unimers (non-stacked conjugates) in solution decreasing the overall intensity. However, the data followed the same trend as already shown from light scattering with a strong increase in *N*_agg_ for DP = 20 and significantly longer tubes for responsive system as compared to non-responsive tubes (DP = 20). Remarkably, for these tubes (**24**), the model had to be changed to a flexible cylindrical micelle (with a Kuhn length of 400 Å) to fit the data appropriately. This suggests an unexpected high flexibility of these supramolecular polymers in water, which could be a result of their high aspect ratio (as compared to the other compounds) in combination with the diminished sterical demand of their polymer shell. A possible explanation for the difference in the length between nanotubes **24** and **25** could be minor variations in conjugation efficiency or DP of the connected polymer. While CPNT with DP = 45 polymers are sufficiently stabilized, the conjugation of PEtOx with a DP of 10 leads to materials that are completely water insoluble due to aggregation. Compounds **24** and **25** are in between these two extremes and even a minor difference within the composition and density of the polymer shell could lead to a significant variation of tubular length. While SEC measurements do not suggest any difference between the two compounds, even low amounts of detached polymer could lead to the observed SANS results. Indeed, the inherent dynamic properties of disulfide connections in solution could lead to a sufficient destabilization of the polymer corona to explain this increased tubular size. This is supported by the smaller size of compound **25**, where the lack of cleavable connections prevents the partial destabilization of CPNT. It should be noted that nanotubes still remain colloidally stable in aqueous solution over the cause of days.

The stimuli responsive detachment of the polymer arms of our CPNT–polymer conjugates was studied using dynamic light scattering (DLS). This method allows direct measurement of the size of nanoscopic objects in solution, and it is ideally suited to investigate the kinetic of the cleaving reaction. Detachment of the polymeric arms was performed by using 1,4-dithiothreitol (DTT) at a concentration of 30 mM. This reducing agent cleaves the disulphide connection and detaches the PEtOx chains from the cyclic peptide, which in turn will lead to the formation of larger agglomerates due to an increased tendency of the CP to stack, as well as to the induction of lateral aggregation between nanotubes.^10^ It should be noted that the radius (*z*-average) is based on assumptions made for spherical objects and is, thus not fully accurate for nanotubes.

**Fig. 2:**
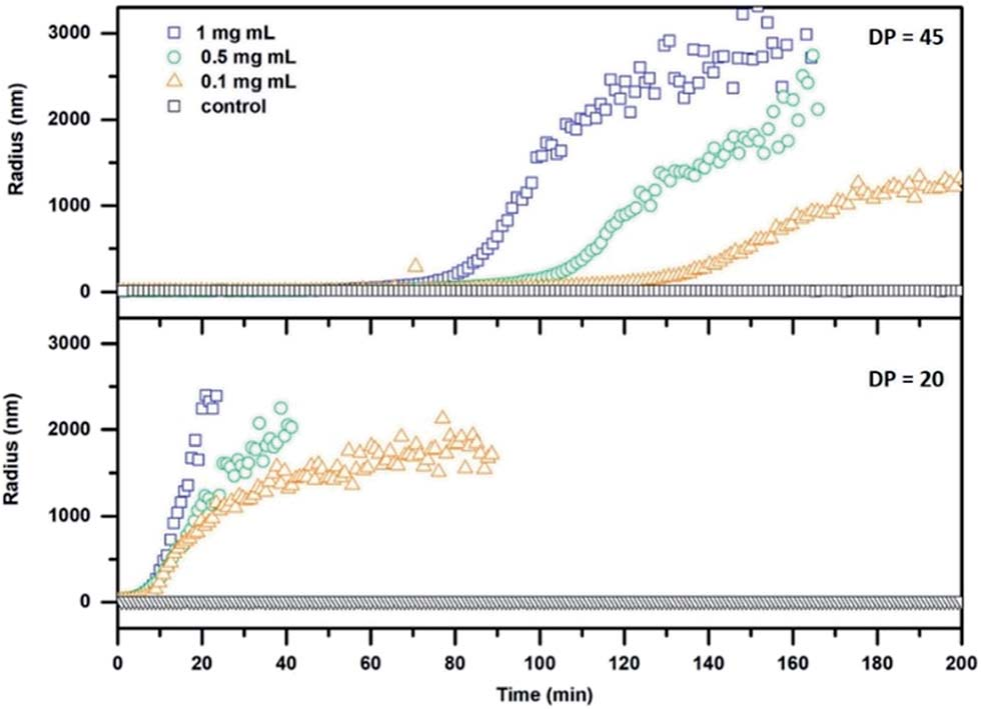
DLS kinetics of cleavable conjugates with a DP of 20 (24; bottom) and 45 (26; top) respectively. DTT at a concentration of 30 mM was added at *t* = 0 min.

The effect of DTT on cleavable CPNT–polymer conjugates is visualized in Fig. 2. Upon the addition of the reduction agent a steady increase in size is observed, after an initial lag-period. In contrast, non-responsive conjugates used as control (black squares in Fig. 2) show a constant hydrodynamic radius in the size range of the intact conjugate.

Additionally, we observe that the size of the polymer attached to the CP in the conjugate plays an important role for the dynamic nature of the system. A pronounced lag phase for conjugates carrying PEtOx polymers with a DP of 45 is observed, whereas an almost immediate response is detected for the DP 20 conjugates. This effect can be ascribed to the increased ability of long polymer arms to stabilize the nanotubes against lateral aggregation. In addition, a thicker polymer shell could potentially shield the cleavable bond from DTT, decreasing the reaction rate. Furthermore, a decrease in concentration of cyclic peptide results in a delayed response of the system at equal DTT concentrations. The system could also be tuned to respond to triggers that are native to cells, such as glutathione (GSH). GSH is an important antioxidant found in cells and acts as a reducing agent, thus making it an optimal target for applications of our system in the biomedical field. A response to GSH at 10 mM (representing maximum naturally occurring concentrations) was tested using the setup described above, but only the DP 20 conjugates (at a concentration of 1 mg mL^–1^) showed aggregation after a time period of 24 h (Fig. S29†). This feature shows that it is possible to create temporally controlled materials (depending on the properties of the reducing agent) that can be triggered depending on specific requirements in a given environment.

The slow kinetics using GSH as a reducing agent is, due to its similar reduction potential to DTT, based on steric effects, which is an important finding for the design of future materials.

These results illustrate the importance of the polymer length on its detachment from the conjugate and the respective kinetics. However, aggregation studies do not provide information on the state of the final material in terms of remaining polymer arms. Furthermore, the number of polymer arms required to detach per tube in order to lead to lateral aggregation is unknown. To elucidate this, mixed systems were produced by the conjugation of a mixture of responsive and non-responsive polymers (**12** and **18**) to the peptide (Scheme S3†). As molecular weight and functionalization efficiency is similar for both polymers, a statistical distribution of both polymers on the CPNT can be expected. Due to the high variability in tubular length for conjugates with PEtOx_20_ arms, DP = 45 polymers were chosen for this study. All conjugates show *N*_agg_ values similar to nanotubes possessing a polymeric shell containing purely responsive or non-responsive chains (as determined by SLS; Fig. S31–35†). Using the above described protocol for detachment of the polymer arms, it was found that when the nanotube shell consisted of 25% detachable arms the addition of 30 mM DTT does not result in either an increase in stacking or aggregation as monitored by a constant *R*_H_ according to DLS experiments (Fig. S36†). Additionally, SLS measurements show only a slight increase in *N*_agg_ after cleavage of disulphide connections (Table S4†). Nanotubes formed using 50% cleavable polymer arms showed a constant *R*_H_ over time (DLS), while SLS measurements after cleavage show a strong increase of *N*_agg_ to around 300. This indicates an increase of stacking in the absence of lateral aggregation after reductive cleaving. However, if 75% of cleavable arms were used to generate the nanotubes, addition of DTT led to aggregation followed by precipitation, indicating the presence of lateral aggregation (Fig. S36†).

While previous studies by Ghadiri and co-workers showed that thiol functionalized CPs do not stack into nanotubes after oxidation,^33^ the reducing environment should in the present case prevent any oxidative covalent coupling and CPs are still expected to form nanotubes after detachment of the polymer. In addition, the preorganization of CPNT–polymer conjugates into long fibrillar structures as detected by SANS measurements should increase this tendency further. However, due to their non-shielded nature a substantial impact of lateral aggregation, especially in the case completely unshielded tubes is expected.

These results show that not only aggregation can be triggered but also tubular length can be adjusted when a sufficient portion of non-active, remaining polymer is installed. As the aspect ratio of such systems can have a significant influence on the interaction with *i.e.* cellular membranes,^34^ this approach represents a further interesting feature of stimuli responsive CPNT–polymer conjugates.

While aggregation studies proved that we could control the stacking of our CPNT systems and form long aggregated nanotubes upon addition of a reducing agent, the permeabilization of lipid bilayers is of paramount importance for our concept. It has been reported that CP form channels or disrupt lipid bilayers and that this process can be influenced by the presence and nature of a polymer shell.^20^ The presence of a hydrophilic PEtOx shell should shield interactions between CPNT and lipid bilayers, while the cleavage of the polymeric shell should lead to increased membrane activity.

This was tested by studying the interaction of our CPNT with synthetic liposomes. A mixture of phosphatidylethanolamine and phosphatidylglycerol was used to simulate the cytosolic membrane of *E. coli*.^35^ Vesicles were loaded with self-quenching concentrations of a fluorescent dye (calcein). This allows to detect disruption of the bilayer, as dye release from the liposomes and the resulting dilution leads to an increase in fluorescence (Scheme S4†).

**Fig. 3:**
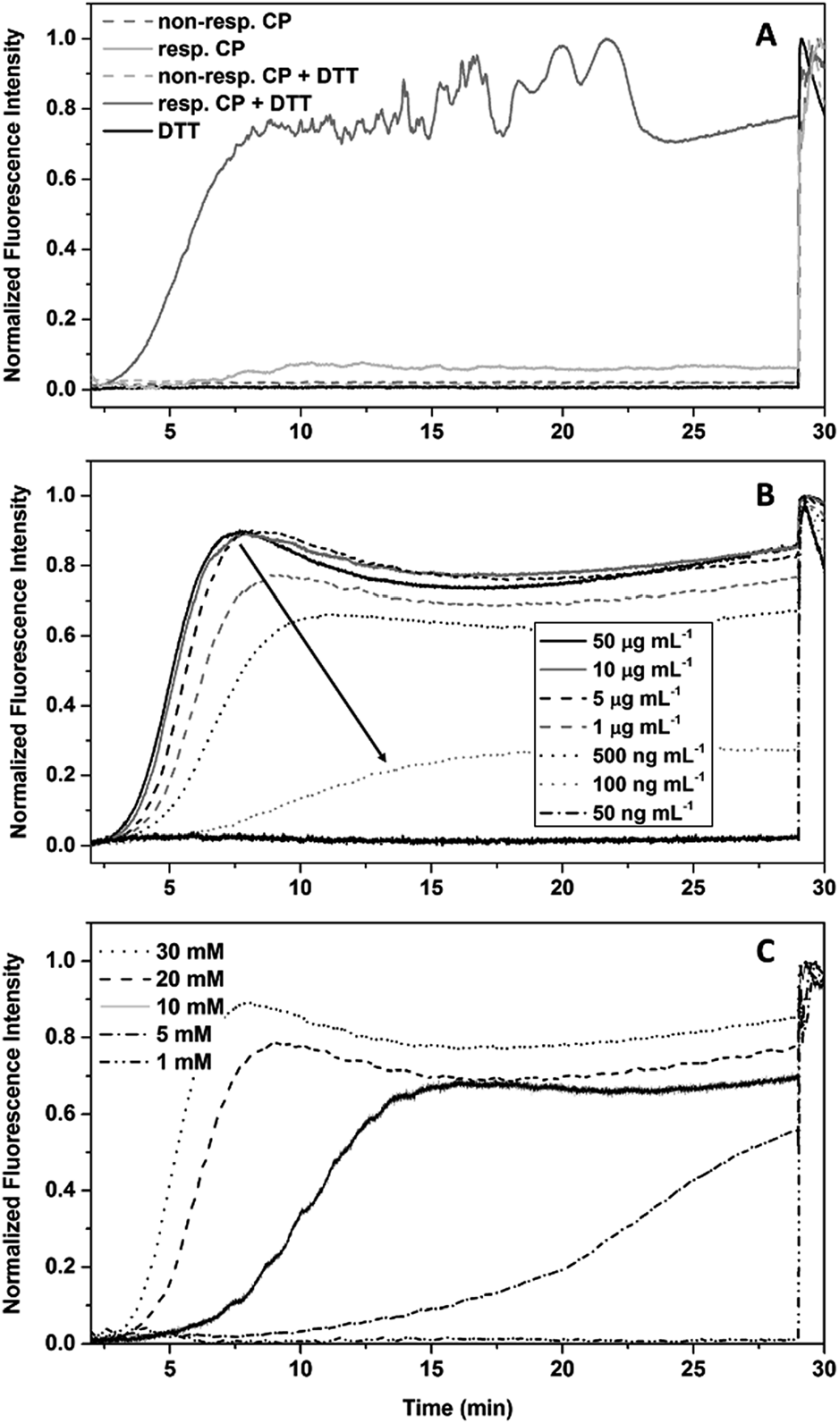
Dye leakage studies of CP–polymer conjugates in the presence of DTT. (A) Proof of concept using responsive (**24**) and non-responsive (**25**) conjugates (1 mg mL^–1^) in the presence and absence of DTT (30 mM), as well as DTT without CPNT. (B) Dependence of the dye leakage on the concentration of conjugate at a DTT concentration of 30 mM. (C) Dependence of the dye leakage on the concentration of DTT at a conjugate concentration of 0.01 mg mL^–1^.

As only DP 20 conjugates show an immediate response to DTT in DLS studies, these compounds were chosen for subsequent dye leakage studies, as long measurement times and the resulting photo-bleaching leads to unreliable results. Both responsive and non-responsive conjugates were used to study their membrane interaction in the presence or absence of DTT (Fig. 3A). As expected, membrane damage, detected as the release of calcein, is only observed when responsive conjugates are treated with DTT. Cleavable conjugates in the absence of DTT, and non-responsive conjugates, shown no effect on liposomes. To exclude membrane damage caused by the reducing agent, a control experiment with pure DTT was undertaken and showed no effect on the fluorescence intensity.

In contrast to DLS measurements, where aggregates are detected, the polymer detachment is visualized indirectly *via* the membrane interaction. Therefore, lower concentrations of conjugates could be investigated.

The ability of compound **24** to disrupt the liposome integrity in presence of DTT depends on the concentration of the CPNT–polymer conjugates used. Comparing the level of fluorescence after DTT addition with the final value (obtained by Triton X addition (1% in water)) allowed us to determine an effective concentration (EC_50_) of 0.21 μg mL^–1^ for liposomal disruption (Fig. 3B). Equally, lower concentrations of DTT led to a retardation of the reduction of the disulphide linkages, leading to higher lag times before liposomal disruption. 5 mM was the lowest concentration of DTT still able to yield membrane disruption. The use of GSH as reducing agent did not lead to liposome membrane disruption within the 30 min time frame of the experiment. Unfortunately, longer experimental times could not be used, due to photo-bleaching of the calcein.

The release of Calcein from vesicles is indicative that the presented system can trigger lipid membrane disruption upon external stimulation. From the gathered information a statement about the mode of interaction (*e.g.* pore formation or random disintegration) is not possible. However, the presence of precipitate shortly after dye release points towards a complete disruption and aggregation of membrane components.

**Fig. 4:**
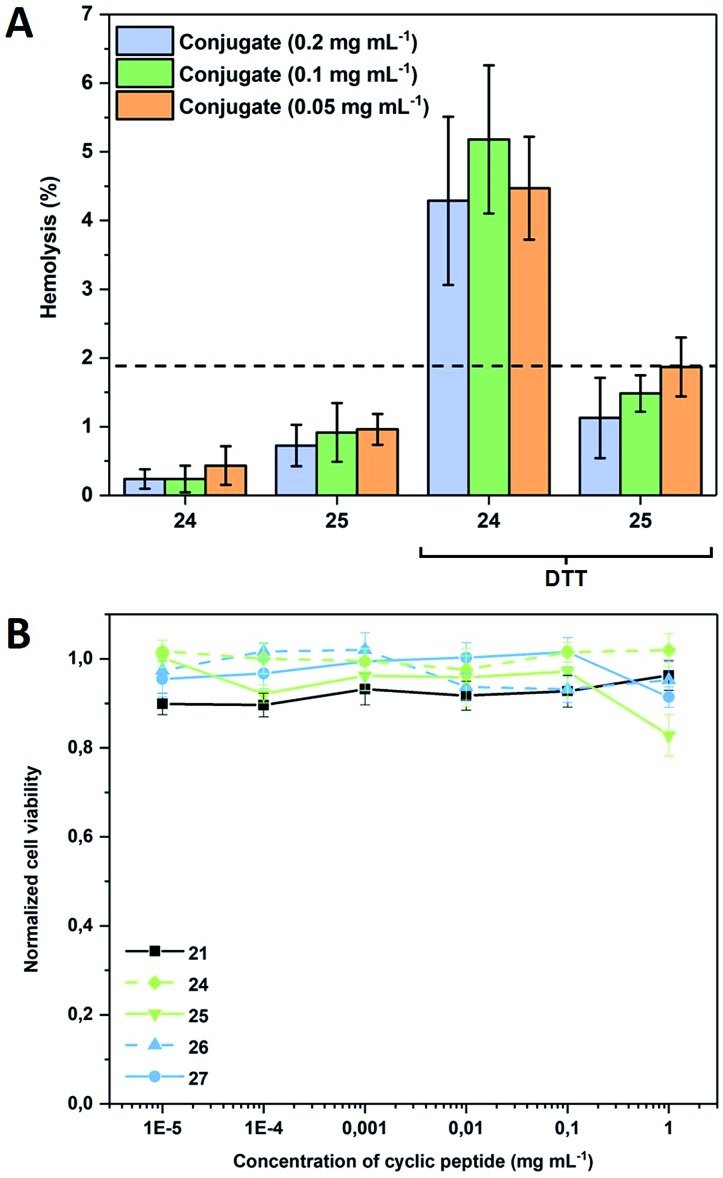
(A) Haemolysis of responsive (**24**) and non-responsive (**25**) CPNT conjugates in the absence and presence of DTT (30 mM). RBCs were obtained from defibrinated donor sheep blood. Measurements were performed after 1 h incubation at 37 °C in duplicates of triplicates; (B) cell viability of Caco2 cells after 72 h of incubation with varying concentrations of cyclic peptide as well as responsive and non-responsive CP–polymer conjugates at 37 °C. Displayed concentration states the amount of cyclic peptide disregarding the polymer shell.

To demonstrate that such system can also be used to permeate naturally occurring membranes, which possess an increased complexity compared to the used models, red blood cells (RBCs) were used (Fig. 4A). As expected, the presence of a PEtOx shell shields the nanotubes sufficiently to maintain haemolysis levels below 2%, which is generally considered as the threshold for haemolytic behaviour (according to the ASTM F756-00 standard). When the same experiment is performed in the presence of DTT, responsive compounds show a concentration independent increase above this level, while non-responsive conjugates remain comparably inactive thus proving that also biological membranes can be triggered by the approach presented herein.

To verify that the activation of the compounds would not lead to a severe systemic effect, cytotoxicity against adherent mammalian cells was also tested and was found to be insignificant for all compounds including the bare CP within a relevant concentration range demonstrating the general biocompatibility of the systems (Fig. 4B). The addition of DTT to the CP-conjugates shortly before subjecting them to cytotoxicity investigations was tested and found to have no effect (data not shown), which is not surprising as the bare CP does not reduce cell viability either.

This approach demonstrates the general possibility of a directed membrane permeabilization using CPNT, with tunable activity time frame. While initial compounds are biocompatible, activation leads to an increased membrane activity, which could be utilized for multiple applications. The use of a reduction sensitive connection is only one example of possible stimuli responsive cyclic peptides able to target specific membranes based on their chemical or biological environment.

## Conclusion and outlook

In conclusion, we have demonstrated the synthesis of stimuli responsive, membrane active cyclic peptide nanotubes (CPNT) polymer conjugates. The approach shown allows us to control the stacking and aggregation of CPNT by means of the presence or absence of a pre-established chemical stimulus. Therefore, it is possible to transform an inert supramolecular polymer into a membrane active substance within seconds. While the initial conjugates, shielded by the polymer shell, remain inactive towards membranes, an on-demand activation of their pore-formation abilities could be generated by the chemically induced detachment of the macromolecules (yielding an increase in tubular length and lateral aggregation). Using different polymers it is also possible to tune the kinetics of the resulting cleavage process enabling to tailor our functional materials for specific purposes. But also tubular length, and hence the aspect ratio of the materials can be tuned *in situ*, by stimuli responsive cleavage of a predefined share of the polymer shell, without causing lateral aggregation, which has potential impact on interactions with surfaces and membranes.

Finally, the designed materials were tested on naturally occurring membranes and shown to be able to permeate them when triggered, while in the absence of a chemical stimulus they remain inert. This, combined with the biocompatibility of these compounds renders them highly interesting for biomedical applications such as drug delivery of antimicrobial purposes.

The herein presented system offers a platform for the design of materials able to serve in specific applications. Future work will focus on linker sequences that can be cleaved by specific (*e.g.* pathogen derived) stimuli and on using a central CP unit with enhanced membrane permeabilization abilities. Using the knowledge about how various parameters influence the dynamics of such conjugates will prove essential for this intend.

## Conflicts of interest

There are no conflicts to declare.

## Supplementary Material

Supplementary information
