# Supplementary material for: Stimuli-responsive membrane activity of cyclic-peptide–polymer conjugates
Source: Chem Sci. 2019 Apr 18;10(21):5476–83. doi: 10.1039/c9sc00756c (PMC6544120; doi:10.1039/c9sc00756c)
Supplement: Supplementary file 1 [file SC-010-C9SC00756C-s001.pdf]

Electronic Supplementary Material (ESI) for Chemical Science.
This journal is © The Royal Society of Chemistry 2019

Supporting Information

Stimuli-Responsive Membrane Activity of Cyclic-Peptide-Polymer Conjugates

*Matthias Hartlieb, Sylvain Catrouillet, Agnès Kuroki, Carlos Sanchez-Cano, Raoul Peltier, Sébastien Perrier**

Dr. M. Hartlieb, Dr. S. Catrouillet, A. Kuroki, Dr. C. Sanchez-Cano, Dr. R. Peltier, Prof. S. Perrier: Department of Chemistry, University of Warwick, Gibbet Hill Road, Coventry CV4 7AL, United Kingdom.

Prof. S. Perrier: Faculty of Pharmacy and Pharmaceutical Sciences, Monash University, 381 Royal Parade, Parkville, VIC 3052, Australia.

Prof. S. Perrier: Warwick Medical School, The University of Warwick, Coventry CV4 7AL, U.K.;

*Corresponding author: s.perrier@warwick.ac.uk

Dr. M. Hartlieb - Current Address: Institute of Chemistry, University of Potsdam, Karl-Liebknecht-Straße 24-25, Potsdam 14476, Germany.

Dr. S. Catrouillet - Current Address: Institut Charles Gerhardt Montpellier UMR5253 CNRS-UM-ENSCM, Université de Montpellier, F-34095, Montpellier Cedex 5, France

Experimental part

Materials and Instrumentation

Chemicals and solvents were purchased from Fisher Scientific, Sigma-Aldrich, Merck, Fluka, and Acros. 2-Ethyl-2-oxazoline (EtOx) and methyl tosylate (MeTos) were distilled to dryness prior to use. EtOx was dried using CaH_2 before distillation. Fmoc protected amino acids and HCTU were obtained from Iris Biotech GmbH (Germany).

^{1}H-NMR spectra were measured using a Bruker DPX-300 or DPX-400 NMR spectrometer which operated at 300.13 and 400.05 MHz, respectively. The residual solvent peaks were used as internal references.

For SEC measurements of polymers in chloroform, an Agilent 390-LC MDS instrument with differential refractive index (DRI), viscometry (VS), dual angle light scatter (LS) and two wavelength UV detectors was used. The system was equipped with 2 x PLgel Mixed D columns (300 x 7.5 mm) and a PLgel 5 μm guard column. The eluent was $CHCl_3$ with 2 % TEA (triethylamine) additive. Samples were run at 1 mL min^{-1} at 30 °C. Poly(methyl meth-acrylate), and polystyrene standards (Agilent Easy Vials) were used for calibration.

Cyclic peptide conjugates were analyzed on a Polymer Laboratories PL-GPC 50 Plus system using a PolarGel-M guard column (7.5 × 50 mm) followed by two PolarGel-M columns (7.5 × 300 mm). DMF (0.1% LiBr) was used as eluent at 1.0 mL min^{-1} at 50 °C. Commercial narrow linear poly(methyl methacrylate) standards in range of 2.0×10^2−1.0×10^6 g mol^{-1} were used to calibrate the DMF SEC system.

Analyte samples were filtered through a nylon membrane with 0.22 μm pore size before injection. Respectively, experimental molar mass (M_n, SEC) and dispersity (*Đ*) values of synthesized polymers were determined by conventional calibration using Agilent GPC/SEC software.

Electrospray Ionisation (ESI) spectra were obtained using a Bruker MicroToF and the results analysed using Bruker Data Analysis. Samples were dissolved in methanol at a concentration of 1 μg mL^{-1}.

The fluorescent intensity was monitored using Agilent Technologies Cary Eclipse Fluorescence Spectrophotometer. The solutions of vesicles were introduced in polystyrene cuvettes for the measurements.

Static light scattering

Light scattering measurements were obtained using an ALV-CGS3 system operating with a vertically polarized laser with wavelength λ = 632 nm. The measurements were taken at 20 °C,

over a range of scattering wave vectors (q = 4πn sin(θ/2)/λ, with θ the angle of observation and n the refractive index of the solvent). The measurements determined the relative excess scattering (I) which is defined as,

$$I = \frac{I_{solution}(\theta) - I_{solvant}(\theta)}{I_{tol}(\theta)} R_{\theta}^{tol} \quad (1)$$

where I_{solution}, I_{solvent} and I_{toluene} are the scattering intensities of the solution, solvent and reference (toluene) respectively, and R_{θ}^{tol} the Rayleigh ratio of toluene (R_{θ}^{tol} = 1.35 x 10^{-5} cm^{-1} for λ = 632.8 nm). I is then expressed in 1/Å and is related to the apparent weight-average molar mass (M_a) as

$$I = KCM_a \quad (2)$$

The optical constant, K, is defined for light scattering by eq. 2,

$$K_{LS} = \frac{4\pi^2 n^2}{\lambda^4 N_A}\left(\frac{\partial n}{\partial C}\right)^2\left(\frac{n_s}{n}\right)^2 \quad (3)$$

where N_a is Avogadro number and dn/dC is the incremental refractive index, n_s is the refractive index of toluene and n of the solvent. (n_{water} = 1.333, n_{toluene} = 1.496).

At a given concentration the Rayleigh ratio, R_θ, is related to the apparent molecular weight of the sample, given by eq. 3. It is only at infinite dilutions, where the interactions between scattering particles are negligible, that the apparent molecular weight is equal to the true molecular weight.[1] Multiple concentrations were measured and a plot of linear regression used to determine the apparent molecular weight at a concentration of 0 mg mL^{-1}.
The incremental refractive index, dn/dC, was determined by measuring the refractive index of the polymer over a range of concentrations. The RI was determined using a Shodex RI detector, operating at a wavelength of 632 nm. Multiplying the gradient, of the plot of RI vs conc., by

the refractive index of the solvent (water = 1.3325) and dividing by the RI constant of the instrument (-1,398,000) gives the d*n*/d*C* of the polymer.

Dynamic light scattering

DLS measurements were conducted on an ALV-CGS3 system at λ = 632 nm, 20 °C and an angle of 120°. After an equilibration time of 180 s, 3 × 30 s runs were carried out at 25 °C. Each measurement was performed in triplicate. Apparent hydrodynamic radii, Rh, were calculated according to the Stokes–Einstein equation. For kinetic investigations of reduction induced polymer cleavage, scattering fluctuation was measured over 30 s. Time points were taken with 30 s intervals over 1 to 6 h.

Small angle neutron scattering (SANS)

Measurements were performed on the SANS Instrument D11 at Institut Laue-Langevin in Grenoble, France. Scattering intensities were recorded by a two-dimensional position-sensitive ^{3}He detector. Three different instrument settings were used corresponding to a momentum transfer range $q = 4\pi \sin\frac{\theta}{2}/\lambda$ of 0.0066 < q < 0.24. H_2O was used for instrumental calibration. The data were placed on an absolute scale (cm^{-1}) using the scattering from a standard sample in accordance with established procedures.[2] In order to compare SANS and light scattering data directly, we have expressed the SANS results in terms of Ma using equation 4. For SANS the constant K is given by:

$$K_{SANS} = \frac{1}{N_A} \times \left(\frac{\rho_{solute} - \rho_{solvent}}{d}\right)^2 \qquad (4)$$

where d is the density of the solution. ρ_{solute} is the scattering length density for the polymer and has been computed according to its chemical structure.

The obtained reduced data was analyzed with the open access software SASfit.[3] For the conjugates 25, 26 and 27 a form factor of a diblock copolymer micelle with rod-like core was applied.[4] The best fit for the conjugate 24 was obtained using a form factor of a micelle with a flexible cylindrical core.[4]

<u>Form factor for a micelle with a rod-like core</u>

The best model used to fit the SANS data for the conjugates 25, 26 and 27 in D_2O was that of a hairy rod-like micelle.

$$P(q) = N^2\beta_s^2 F_{s(q)} + N\beta_c^2 F_{c(q)} + 2N^2\beta_s\beta_c S_{sc(q)} + N(N-1)\beta_c^2 S_{cc(q)} \quad (5)$$

where N is the aggregation number, $\beta_s = V_s(\rho_s - \rho_{solv})$ and $\beta_c = V_c(\rho_c - \rho_{solv})$ are the total excess scattering lengths of a block in the cylindrical core and in the corona, respectively. V_s and V_c are the volumes of a block in the core and in the corona, respectively. ρ_s and ρ_c are the corresponding scattering length densities and ρ_{solv} is the scattering length density of the surrounding solvent.

$F_{s(q,R,L)} = F_{cs(q,R)} F_{L(q,L)}$ where $F_{cs(q,R)} = \left[\frac{2B_1(qR)}{qR}\right]^2$, $F_{L(q,L)} = 2\frac{Si(qL)}{qL} - \frac{4sin^2\left(\frac{qL}{2}\right)}{q^2L^2}$ B_1 is the first order Bessel function and $Si(x) = \int_0^x \frac{\sin t}{t}dt$

$$F_{c(q,Rg)} = \frac{2\left[\exp\left(-q^2R_g^2\right) - 1 + q^2R_g^2\right]}{q^4R_g^4}$$

$S_{sc(q,R,Rg,L)} = \psi(qR_g)\frac{2B_1(qR)}{qR}B_0[q(R + dR_g)]F_{L(q,L)}$ where $\psi(qR_g) = \frac{1 - exp(-qR_g)}{qR_g}$, R_g is the gyration radius of the block of the corona, and B_0 is the zero[th] order Bessel function.

$S_{cc(q,R,Rg,L)} = \psi(qR_g)^2B_0[q(R + dR_g)]^2F_{L(q,L)}$.

d was set to 1.

The fit (Figure S26, S27 and S28) were performed with R, L, Rg, N_{agg} and N as adjustable parameter. The values afforded by the fits are gathered in Table S1 in the following.

Table S1. Fitting parameters for 25,26 and 27 using a form factor of a micelle with a rod-like core

**Parameter**	**25**	**26**	**27**	
R_{core} (Å)	6.4	6.4	6.4	Fitting parameters
L_{core} (Å)	270	67	59	
$R_{g,\ corona}$[a] (Å)	18	22	22	
N_{agg}[b]	58	14	13	
N	0.25	58300	70000	
V_{core} (single unit)[c] (Å^3)	300	300	300	Calculated parameters
V_{corona} (single unit)[c] (Å^3)	3170	5530	5810	

ρ_{core}^{d} (cm^{-1})	9.45×10^{-7}	1.10×10^{-7}	1.10×10^{-7}
ρ_{corona}^{d} (cm^{-1})	9.45×10^{-7}	1.10×10^{-7}	1.10×10^{-7}
$\rho_{solvent}^{d}$ (cm^{-1})	6.38×10^{-6}	6.38×10^{-6}	6.38×10^{-6}
d^{e}	1	1	1
Background subtracted (cm^{-1})	0.05	0.05	0.05

[a] Radius of gyration (R_g) of a single chain in the corona;

[b] The number of aggregation (N_{agg}) was calculated from the volume of the whole core divided by the volume of the core of a single unit;

[c] The Volume of a single cyclic peptide (Vcore) in the core or polymer corona (PEtOx, Vcorona) was estimated from the M_n, divided by the Avogadro constant and assuming a density of 1 g/cm^3,

[d] The scattering lengths density (SLD) of the materials was calculated using the calculator given in SASfit;

[e] d represents a penetration factor for chains of the brush entering the core with no penetration giving a value of 1. To account for the rigid CP nanotube at the interface it was set to 1.1.

Form factor for a micelle with a flexible cylindrical core

From equation 5, a flexible cylindrical core was described using Kholodenkos approach to reproduce the rigid rod limit and the random-coil limit. Defining $x = 3L/l$ (L: contour length, l: Kuhn Length) and adapting $F_{s(q,R,L,l)}$, $S_{SC(q,R,L,l)}$, $S_{CC(q,R,L,l)}$ by:

$$F_{s(q,R,L,l)} = P_{worm(q,L,l)} \times P_{cs(q,R)} \text{ with } P_{cs(q,R)} = \left[\frac{2B_1(qR)}{(qR)}\right]^2 \text{ and } P_{worm(q,L,l)} = \frac{2}{x}\left[I_1 - \frac{1}{x}I_2\right]$$

where
$$I_{(n)}(x) = \int_0^x f(z) z^{n-1} dz$$

together with
$$f(z) = \begin{cases} \dfrac{1}{E}\dfrac{\sinh(Ez)}{\sinh(z)} & for\ Q \le \dfrac{3}{l} \\ \dfrac{1}{F}\dfrac{\sinh(Fz)}{\sinh(z)} & for\ Q \ge \dfrac{3}{l} \end{cases}$$

and $E = \sqrt{1 - \left(\frac{lQ}{3}\right)^2}$ and $F = \sqrt{\left(\frac{lQ}{3}\right)^2 - 1}$

$$S_{sc(q,R,Rg,L,l)} = \psi(qR_g)\frac{2B_1(qR)}{qR}B_0\left[q\left(R + dR_g\right)\right]P_{worm(q,L,l)}$$

$$S_{cc(q,R,Rg,L,l)} = \psi(qR_g)^2 B_0\left[q\left(R + dR_g\right)\right]^2 P_{worm(q,L,l)}$$

The following values were estimated from the scattering length densities:

$\rho_{solv} = 6.38\ 10^{-6}$ Å^{-2}, $\rho_s = 9.45\ 10^{-7}$ Å^{-2}, $\rho_c = 9.45\ 10^{-7}$ Å^{-2}

The fit (Figure S25) was performed with R, Rg, N_{agg}, l and N as adjustable parameter. The values afforded by the fit are gathered in Table S2 in the following.

Table S2. Fitting parameters for 24 using a form factor of a micelle with a spherical core

**parameter**	**24**		
N_{agg}	1900	Å	
R_{core}^{a}	6.4	Å	Fitting
$R_{g,\ corona}^{b}$	16.6	Å	parameters
N	6155		
l	400	Å	
V_{core} (single unit)^{c}	300	Å^3	
V_{corona} (single unit)^{c}	3320	Å^3	
ρ_{core}^{d}	9.45×10^{-7}	cm^{-1}	Calculated
ρ_{corona}^{d}	9.45×10^{-7}	cm^{-1}	parameters
$\rho_{solvent}^{d}$	6.38×10^{-6}	cm^{-1}	
Background subtracted	5×10^{-2}	cm^{-1}	

[a] The radius of the core was calculated from the volume of the whole core given by the volume of the core of a single unit multiplied with the number of aggregation (N_{agg});

[b] Radius of gyration (R_g) of a single chain in the corona;

[c] The Volume of a single cyclic peptide in the core or polymer corona (PEtOx) was estimated from the M_n, divided by the Avogadro constant and assuming a density of 1 g/cm^3,

[d] The scattering lengths density (SLD) of the materials was calculated using the calculator given in SASfit;

Dye leakage assays

Formation of vesicles. The synthesis of vesicles was performed according to a protocol detailed by Lienkamp *et al.*.[5] 100 mL of a first buffer (buffer A) was prepared by dissolving 142 mg (1 mmol) of Na_2HPO_4 in 90 mL of H_2O. The pH was then adjusted to 7 with a 1 mol.L^{-1} solution of NaOH. The total volume of the solution was then taken to 100 mL. The calcein solution was obtained by dissolving 249 mg (0.4 mmol) of calcein dye in 8 mL of previously prepared buffer A. The pH of the solution was adjusted to 7.0 with a 1 mol.L^{-1} solution of NaOH in order to dissolve the calcein. The total volume was then taken up to 10 mL in order to yield a buffer of 40 mmol.L^{-1} of calcein.

A second buffer (buffer B) was prepared by dissolving 1.42 g (10 mmol) of Na_2HPO_4 and 5.26 g (90 mmol) of NaCl in 980 mL of H_2O. The pH was adjusted to 7.0 with a 1 mol.L-1 solution of NaOH. The volume of the solution was then taken up to 1000 mL.

For the phosphatidylethanolamine (PE) and phosphatidylglycerol (PG) vesicles, 6.0 mg (8 mmol) of PE and 1.6 mg (2 mmol) of PG were dissolved in 0.8 mL of $CHCl_3$, in a 25-mL round bottom flask, in order to obtain a solution of roughly 10 mg.mL^{-1}. A film was formed at the bottom of the flask by removing the solvent under reduced pressure, the flask kept as vertical as possible. After the film was dried under vacuum, it was hydrated with 1 mL of buffer A and stirred for an hour with a magnetic stirring bar. After complete dissolution of the lipid, the solution underwent 5 freeze-thaw cycles. The solution was then filtered 15 times by extrusion, using 400 nm membranes. The free dye was filtered through a Sephadex G-50 column using buffer B. The vesicle fraction from the column was diluted for the dye-leakage experiments according to the initial fluorescence of the solution.

Fluorescence monitoring.

Interactions of the polymers with model bacterial membranes composed of lipid bilayers were evaluated using liposomes consisting of a mixture of PE and PG with a ratio of 4 to 1 to model Gram-negative bacteria. The fluorescent dye calcein was encapsulated in a self-quenching concentration. When the membrane is compromised by the addition of a sample, the dye leakage would result in an increased fluorescence.
To that end the fluorescence of the vesicle solution was monitored by recording the fluorescence intensity at a wavelength of 537 nm with the excitation wavelength set at 492 nm. The intensity of the vesicle solution mixed with the cyclic peptide was measured, then a solution of DTT was added 30 seconds after the start of the run to reach a final concentration of 30 mM of DTT, followed by the addition of 20 µL of a 20 % solution of Triton X 29 minutes later. The intensities were normalized by setting the baseline at the intensity before polymer addition and the maximum at the intensity reached after addition of Triton X, corresponding to 100 % leakage. EC_{50} values were determined using a Hill equation.

Hemolysis assay

Defibrinated donor sheep blood was purchased from Thermo Fisher and red blood cells (RBC) were purified as follows. 2 mL of blood was distributed over Eppendorf tubes and centrifuged at 4500 rpm for 1 min. The supernatant was removed and the remaining RBCs were diluted with 1 mL of sterile PBS (pH 7.4). After mixing the mixture was centrifuged again and the supernatant was removed. The process was repeated until the supernatant remained colorless. RBC were diluted 1:150 in PBS. In Eppendorf tubes, 20 µL of compound solution (at a 20-fold

of the final concentration) was mixed with 380 μL of purified RBC solution. The mixture was incubated for 1 h at 37°C and the RBC were subsequently removed by centrifugation as described above. In the case of DTT addition, a final concentration of 30 mM was added. The absorbance of the remaining solution was measured at 414 nm to determine hemolysis levels. PBS was used as negative control and a 1% solution of triton X-100 was used to lyse all RBCs.

Cell Culture

CaCo2 human colorectal adenocarcinoma cells were grown in a 50:50 mixture of Ham's F12 and DMEM medium supplemented with 10% of fetal calf serum, 1% of 2 mM glutamine and 1% penicillin/streptomycin. They were grown as adherent monolayers at 310 K in a 5% CO2 humidified atmosphere and passaged at approximately at 70-80% confluence.

In vitro growth inhibition assays

The antiproliferative activity of the cyclic peptides carrying or not different polymeric arms was determined in CaCo2 colorectal cancer cells. Briefly, 96-well plates were used to seed 10000 cells per well. The plates were left to pre-incubate with drug-free medium at 310 K for 24 h before adding different concentrations of the compounds to be tested (1 mg ml^{-1}-10 ng ml^{-1}). A drug exposure period of 72 h was allowed. The SRB assay was used to determine cell viability.[6] The experiment was performed as duplicates of triplicates in two independent sets of experiments and their standard deviations were calculated.

Synthesis

*Ethyl xanthate functionalized poly(2-ethyl-2-oxazoline)s **(1 - 3)***

Dry ethyl oxazoline (EtOx), methyl tosylate (MeTos) and acetonitrile were added to a Schlenk flask under nitrogen and left to stir in an oil bath at 78 °C. After a predetermined time, the solution was removed from the oil bath and potassium ethyl xanthate was added to terminate the polymer chain. The mixture was left to stir for 2 h at room temperature. Chloroform (100 mL) was added and the organic layer was then washed with saturated aqueous Na_2CO_3 solution (3 x 100 mL) and brine (3 x 100 mL) then dried over $MgSO_4$. The chloroform was removed under reduced pressure and the polymer was dried under vacuum.

Sample	Target DP	EtOx (mL)	MeTos (µL)	Acetonitrile (mL)	Ethyl Xanthate (mg)	Time (min)
**1**	10	10.1	1509	13.4	1923	55
**2**	20	10.1	755	14.2	962	111
**3**	45	10.1	335	14,6	426	255

^{1}H NMR (**1**, 400 MHz, $CDCl_3$) δ ppm: 3.75 – 3.13 (m, 168 H, backbone), 3,10 - 2.92 (m, 3 H, Methyl group (α-end)), 2.54 - 2.13 (m, 87 H, CH_2 side chain), 1.44 (t, 2.7 H, Methyl group (xanthate)), 1.23 – 0.98 (m, 124 H, CH_3 side chain);

SEC ($CHCl_3$, trimethylamine, PS calibration): M_n = 1,000 g mol^{-1}, $Đ$ = 1.20;

ESI-ToF (**1**): measured: 1149.665 m/z ($M+Na^+$), simulated: 1149.681 m/z;

*Pyridyl sulphide activated poly(2-ethyl-2-oxazoline)s (**4- 6**)*

Xanthate functionalized precursor polymer **1-3** (1 g) was mixed with Dipyridyl sulphide (DPS) (80 mg, 2 eq) in a round bottom flask and degassed using nitrogen. Dimethylamine (33% in Ethanol, 20 mL) was degassed with nitrogen and added to the polymer under stirring. The mixture was stirred at 40°C for 2 h and additional 180 mg of DPS (4 eq) were added. After 1 h at room temperature, the solution was poured into 100 ml of water, which was extracted with chloroform (5 x 50 mL). The organic phases were combined and the solvent was removed under reduced pressure. The residual oily substance was precipitated in ice-cold diethyl ether (2 x 300 mL) and the polymer was obtained as a slightly yellow powder.

^{1}H NMR (**4**, 400 MHz, $CDCl_3$) δ ppm: 8.49 (s, 1 H, pyridyl), 7.68 (m, 2 H, pyridyl), 7.14 (s, 1 H, pyridyl), 3.79 – 3.16 (m, 32 H, backbone), 3.11 -2.73 (m, 5 H, Methyl group (α-end) and CH_2-CH_2-S), 2.55 – 2.09 (m, 16 H, CH_2 (side chain)), 1.27 – 0.93 (m, 24 H, CH_3 side chain);

ESI-ToF (**4**): measured: 1179.696 m/z ($M+Na^+$), simulated: 1170.670 m/z;

SEC ($CHCl_3$, trimethylamine, PS calibration): M_n = 1,000 g mol^{-1}, $Đ$ = 1.20;

*Responsive poly(2-ethyl-2-oxazoline)s with carboxylic acid end groups (**7-9**)*

Pyridyl sulfide protected polymer **4-6** (800 mg, 0.72 mmol) was dissolved in water (20 mL) and an excess of 3-thio propionic acid (1.5 g, 14.54 mol, 20 eq.) was added. The mixture was left to stir at room temperature for 2 h and subsequently extracted with chloroform (5 x 50 mL). The organic phases were combined and the amount of solvent was reduced under reduced

pressure. The polymer was precipitated in cold diethyl ether (100 mL), dried under vacuum and obtained as a white powder.

^{1}H NMR (**7**, 400 MHz, $CDCl_3$) δ ppm: 3.69 – 3.34 (m, 32 H, backbone), 3.10 -2.63 (m, 9 H, Methyl group (α-end), CH_2-**CH_2**-S-**CH_2**-**CH_2**-COOH), 2.54 – 2.20 (m, 16 H, CH_2 (side chain)), 1.24 – 1.00 (m, 24 H, CH_3 side chain);

ESI-ToF (**7**): measured: 1165.681 m/z ($M+Na^+$), simulated: 1165.666 m/z;

SEC ($CHCl_3$, trimethylamine, PS calibration): M_n = 1,300 g mol^{-1}, $Đ$ = 1.09;

*NHS activated responsive poly(2-ethyl-2-oxazoline)s **(10 - 12)***

460 mg of polymer **7-9** (0.46 mmol) were dissolved in 3 mL of dichloromethane and N,N′-Dicyclohexylcarbodiimid (DCC, 104 mg, 0.506 mmol, 1.1 eq.) as well as N-hydroxy succinimide (NHS, 58 mg, 0.506 mmol, 1.1 eq.) were added. The mixture was stirred at room temperature for 24 h and the precipitated was filtered off. The solution was precipitated in 200 mL of cold diethyl ether and the precipitate was filtered off and dried. The polymer was obtained as a white powder.

^{1}H NMR (**10)**, 400 MHz, $CDCl_3$) δ ppm: 3.65 – 3.34 (m, 32 H, backbone), 3.14 -2.76 (m, 13 H, Methyl group (α-end), CH_2-**CH_2**-S-**CH_2**-**CH_2**-CONHS, CH_2 (NHS)), 2.49 – 2.20 (m, 16 H, CH_2 (side chain)), 1.19 – 1.02 (m, 24 H, CH_3 side chain);

ESI-ToF (**10**): measured: 1179.563 m/z ($M+K^+$), simulated: 1179.666 m/z;

SEC ($CHCl_3$, trimethylamine, PS calibration): M_n = 1,400 g mol^{-1}, $Đ$ = 1.11;

*Non-responsive poly(2-ethyl-2-oxazoline)s with carboxylic acid end groups **(13 - 15)***

Xanthate functionalized precursor polymer **1-3** (1 g) was degassed in a flask (by repeating vacuum/N_2 cycles). Dimethylamine (33% in Ethanol, 20 mL) was degassed with nitrogen and added to the polymer under stirring. The mixture was stirred at 40°C for 3 h and subsequently a degassed solution of acrylic acid (30 mL), triethylmine (30 mL) in ethanol (100 mL) were added. The mixture was stirred at room temperature for 24 h and the solvent was evaporated under reduced pressure. The residual mixture was dissolved in 5 mL of dichloromethane and precipitated in 400 mL of cold diethyl ether. The precipitate was collected and re-dissolved in dichloromethane (5 mL). The solution was added dropwise to acetone (300 mL) to precipitate the Michael-addition side product between dimethyl amine and acrylic acid. Acetone was then removed under reduced pressure and the polymer was dissolved in dichloromethane (100 mL) and extracted with water (3 x 20 mL). The solvent was removed and the polymer was obtained as a white powder.

^{1}H NMR (**13**), 400 MHz, $CDCl_3$) δ ppm: 3.62 – 3.38 (m, 32 H, backbone), 3.10 -2.93 (m, 3 H, Methyl group (α-end)), 2.90 – 2.57 (m, 6 H, CH_2-**CH_2**-S-**CH_2**-**CH_2**-COOH), 2.56 – 2.10 (m, 16 H, CH_2 (side chain)), 1.22 – 0.99 (m, 24 H, CH_3 side chain);

ESI-ToF (**13**): measured: 1133.716 m/z ($M+Na^+$), simulated: 1133.693 m/z;

SEC ($CHCl_3$, trimethylamine, PS calibration): M_n = 1,600 g mol^{-1}, $Đ$ = 1.08;

*NHS activated non-responsive poly(2-ethyl-2-oxazoline)s (**16** - **18**)*

The NHS activation protocol was identical to the procedure used for responsive polymers.

^{1}H NMR (**16**), 400 MHz, $CDCl_3$) δ ppm: 3.61 – 3.27 (m, 32 H, backbone), 3.10 -2.68 (m, 13 H, Methyl group (α-end), CH_2-**CH_2**-S-**CH_2**-**CH_2**-CONHS, CH_2 (NHS)), 2.50 – 2.19 (m, 16 H, CH_2 (side chain)), 1.22 – 1.00 (m, 24 H, CH_3 side chain);

ESI-ToF (**16**): measured: 1133.592 m/z ($M+K^+$), simulated: 1132.656 m/z;

SEC ($CHCl_3$, trimethylamine, PS calibration): M_n = 1,200 g mol^{-1}, $Đ$ = 1.09;

*Solid phase peptide synthesis (**19**)*

Linear peptide was synthesised *via* fully automatic Solid Phase Peptide Synthesis (SPPS) in a DMF/DCM solvent system using Fmoc protected amino acids on a 2-Chlorotrityl resin (1.1 mmol g^{-1} loading capacity). The concentration of amino acids during coupling was 0.2 mol L^{-1} and HCTU/*N*-methyl morpholine (NMM) (0.2 mol L^{-1}/0.4 mol L^{-1}) was used as activation agent. The Fmoc deprotection was conducted using 20% piperidine in DMF. Cleavage of protected peptides from the resin was carried out using a 20% HFIP in DCM solution. The cleavage mixture was evaporated under reduced pressure and the linear peptide was dried under vacuum.

^{1}H-NMR (**19**, 300 MHz, TFA-d,): δ (ppm) = 8.08-8.00 (m, 2 H, Trp (C-CH-N), 7.53-7.15 (m, 8 H, Trp (arom. protons)), 5.11-4.98 (m, 2 H, Trp (peptide backbone)), 4.60-4.34 (m, 4 H, (Leu peptide backbone), 4.16 (t, 1 H, Lys (peptide backbone, N-terminus)), 3.27-2.99 (m, 8 H, (Lys (CH_2-CH_2-NHBoc); Trp (CH_2))), 2.05-1.89 (m, 4 H, Lys (CH_2-CH_2-CH_2NR)), 1.83-1.70 (q, 4 H, Lys (CH_2-CH_2-CH_2)), 1.60-1.40 (m, 38 H, Boc (CH_3); Lys (CH-CH_2-CH_2)), 1.28-1.04 (m, Leu (CH_2)) , 1.03-0.78 (m, 4 H, Leu (CH_2-CH-$(CH_3)_2$), 0.78-0.46 (m, 24 H, Leu, CH_3).

ESI-ToF-MS (MeOH, **19**): measured: 1499.902 m/z ($M+Na^+$), simulated: 1499.892 m/z;

*Cyclization of linear peptide (**20**)*

Linear peptide (**19**, 560 mg, 0.36 mmol) was dissolved in DMF (400 mL) and 4-(4,6-Dimethoxy-1,3,5-triazin-2-yl)-4-methylmorpholinium tetrafluoro borate (DMTMM · BF_4)

(141.6 mg, 0.43 mmol, 1,2 eq.) dissolved in DMF (10 mL) was added under stirring. The mixture was left to stir at room temperature for 7 d. The solvent was evaporated under reduced pressure and the residual peptide was suspended in MeOH (100 mL). The compound was isolated by centrifugation and washed with MeOH three times using the same procedure. The cyclic peptide was dried under vacuum and obtained as a white solid (**20**, 250 mg, 45%)

^{1}H-NMR (**20**, 300 MHz, TFA-d): δ (ppm) 8.36-8.31 (m, 2 H, Trp (C-CH-N), 7.69-7.11 (m, 8 H, Trp (arom. protons)), 5.32-5.00 (m, 2 H, Trp (peptide backbone)), 4.83-4.40 (m, 6 H, (Lys/Leu peptide backbone), 3.36-2.80 (m, 8 H, (Lys (CH_2-CH_2-NHBoc); Trp (CH_2))), 1.92 - 1.60 (m, 4 H, Lys (CH_2-CH_2-CH_2NR)), 1.60-1.36 (q, 4 H, Lys (CH_2-CH_2-CH_2)), 1.36-0.44 (m, 74 H, Boc (CH_3), Lys (CH-CH_2-CH_2) (m, Leu (CH_2), Leu (CH_2-CH-(CH_3)$_2$), (Leu, CH_3)).

ESI-ToF-MS (**20**): measured: 1503.892 m/z (M+H$^+$), simulated: 1503.884 m/z;

Deprotection of cyclic peptide (21)

Cyclic peptide (**20**, 200 mg, 0.13 mmol) was dissolved in a mixture of TFA (9 mL), Triisopropyl silane (TIPS) (0.5 mL) and water (0.5 mL) and stirred at room temperature for 2 h. The peptide was precipitated in diethyl ether (100 mL) and isolated by centrifugation. The compound was washed with diethyl ether (2 x 100 mL) and dried under vacuum to yield cyclic deprotected peptide (**21**, 140 mg, 95%)

^{1}H-NMR (**21**, 300 MHz, TFA-d): δ (ppm) = 7.70-6.92 (m, 5 H, Trp (arom. protons)), 6.73-6.53 (s, 5 H (amine)), 5.18-4.95 (m, 2 H, Trp (peptide backbone)), 4.70-4.36 (m, 5 H, (Leu peptide backbone), 3.32-2.95 (m, 8 H, (Lys (CH_2-CH_2-NHBoc); Trp (CH_2))), 1.68-1.56 (m, 12 H, Lys (CH_2)), 1.48-1.04 ((6 H, m, Leu (CH_2)) , 1.04-0.53 (m, 28 H, Leu (CH_2-CH-(CH_3)$_2$), (m, 24 H, Leu, CH_3).

ESI-ToF-MS (**21**): measured: 1081.690 m/z (M+H$^+$), simulated: 1081.692 m/z;

Conjugation of cyclic peptide and NHS activated polymers

For polymer conjugation, cyclic peptide (**21**, 52 mg, 0.039 mmol) was dissolved in DMF (1 mL) and NMM (11 mg, 0.11 mmol, 2.5 eq.) was added. The solution was stirred for 30 min at room temperature and NHS functionalized polymer (0.099 mmol. 2.5 eq.) was added to the mixture. After 3 d the reaction mixture was diluted to 25 mL using water and the conjugate was isolated using centrifuge filter tubes (Amicon, Ultracel – 10K). After freeze drying the product was obtained as a white powder.

Yields: 53% (**22**), 51% (**23**), 60% (**24**), 82% (**25**) 46% (**26**), 38% (**27**).

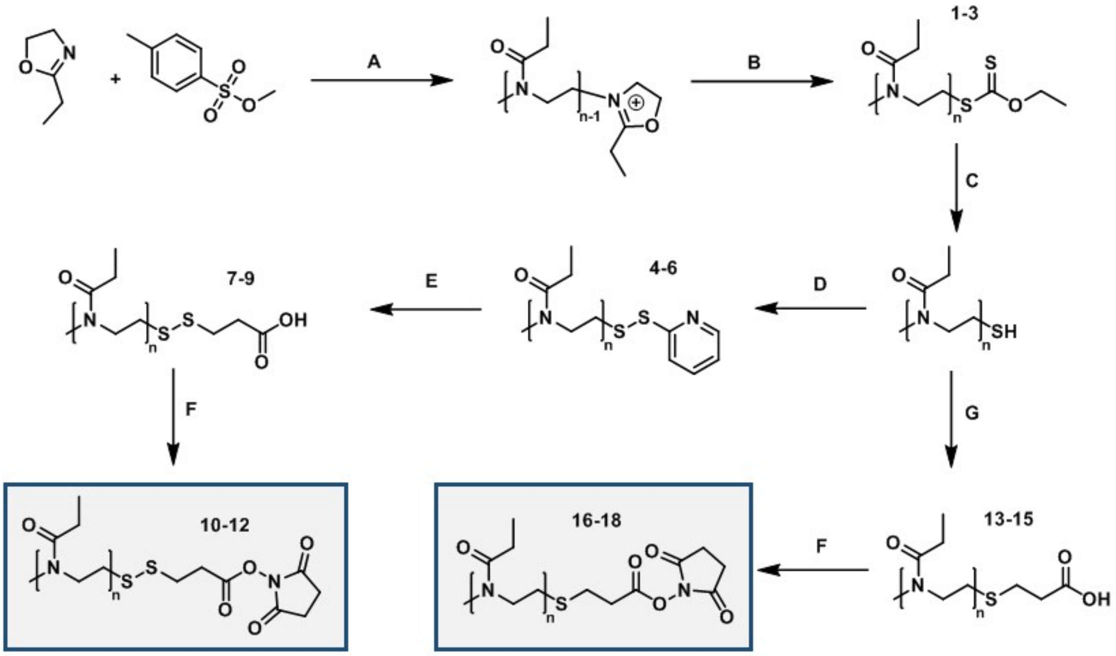

Scheme S1: Synthetic path for responsive and non-responsive Poly(2-oxazoline)s. A) 78˚C, Acetonitrile, varying reaction times; B) Ethyl xanthate, 20˚C, 20 h; C) Dimethyl amine, ethanol, 40˚C, 2 h; D) Dipyridyl sulphide, ethanol 20˚C, 2 h; E) Thiopropionic acid, chloroform, 20˚C, 3 h; F) N-Hydroxy succinimide, dicylcohexyl carbodiimide, dichloromethane, 20˚C, 20 h; G) Acrylic acid, trimethylamine, ethanol, 20˚C, 3 h.

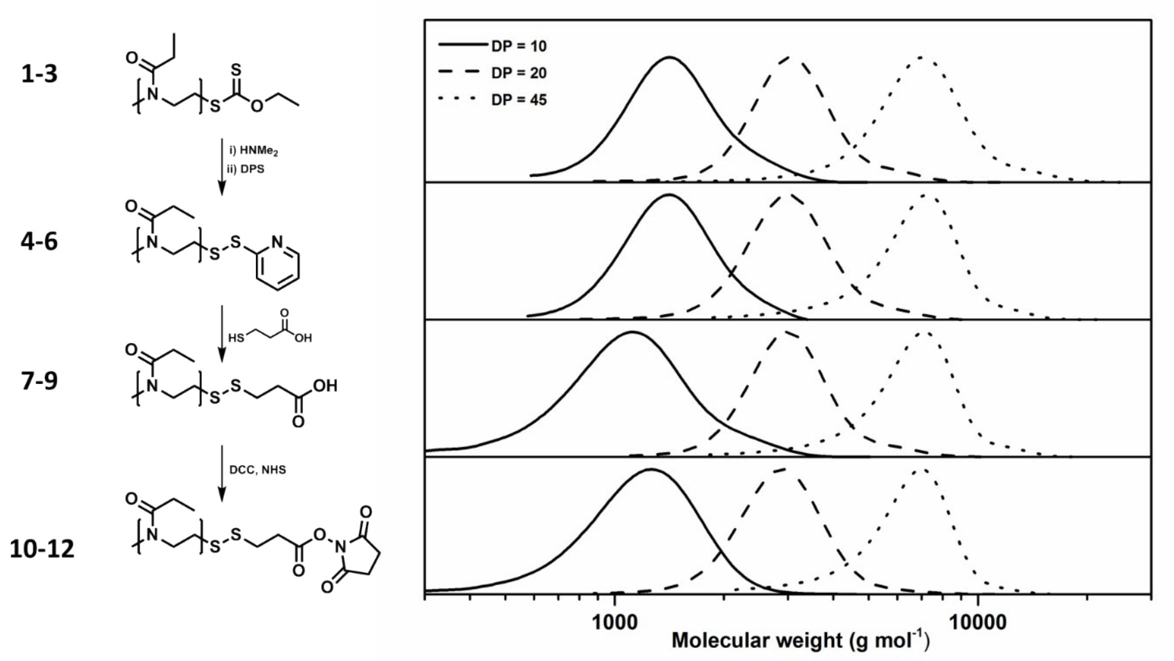

Figure S1: SEC characterization of responsive polymers with different DP values.

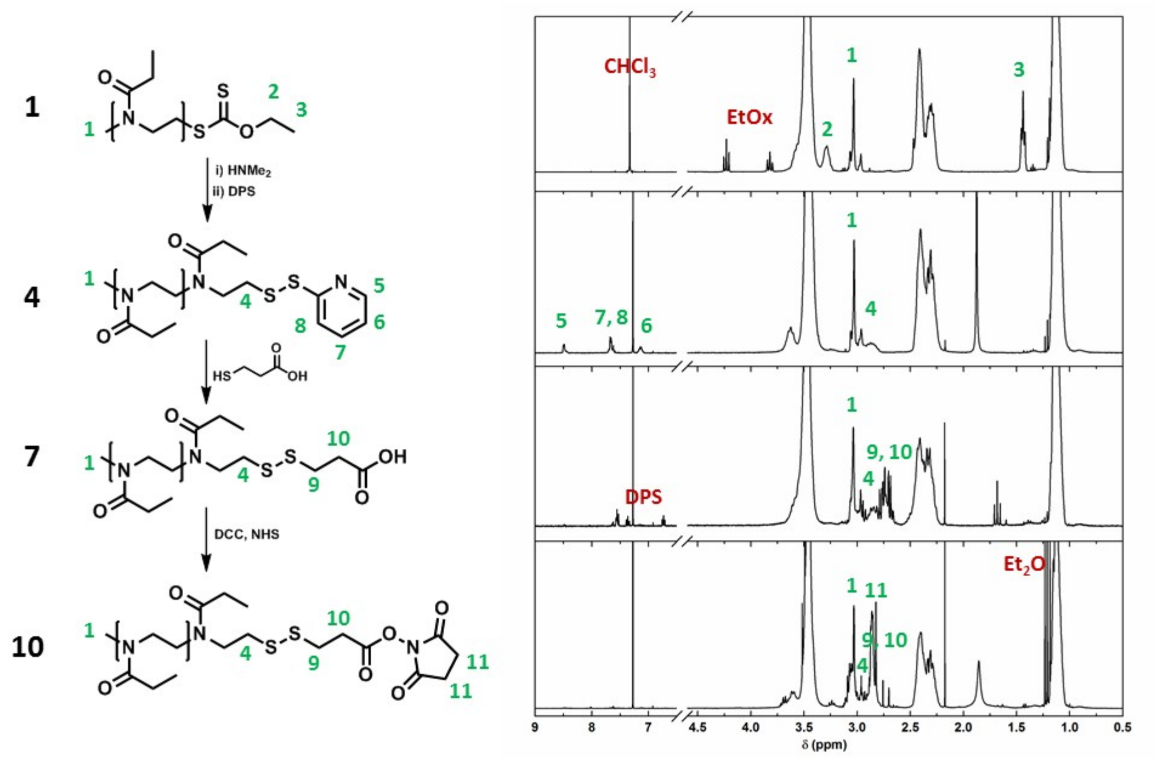

Figure S2: ^{1}H-NMR characterization of responsive polymers with DP = 10.

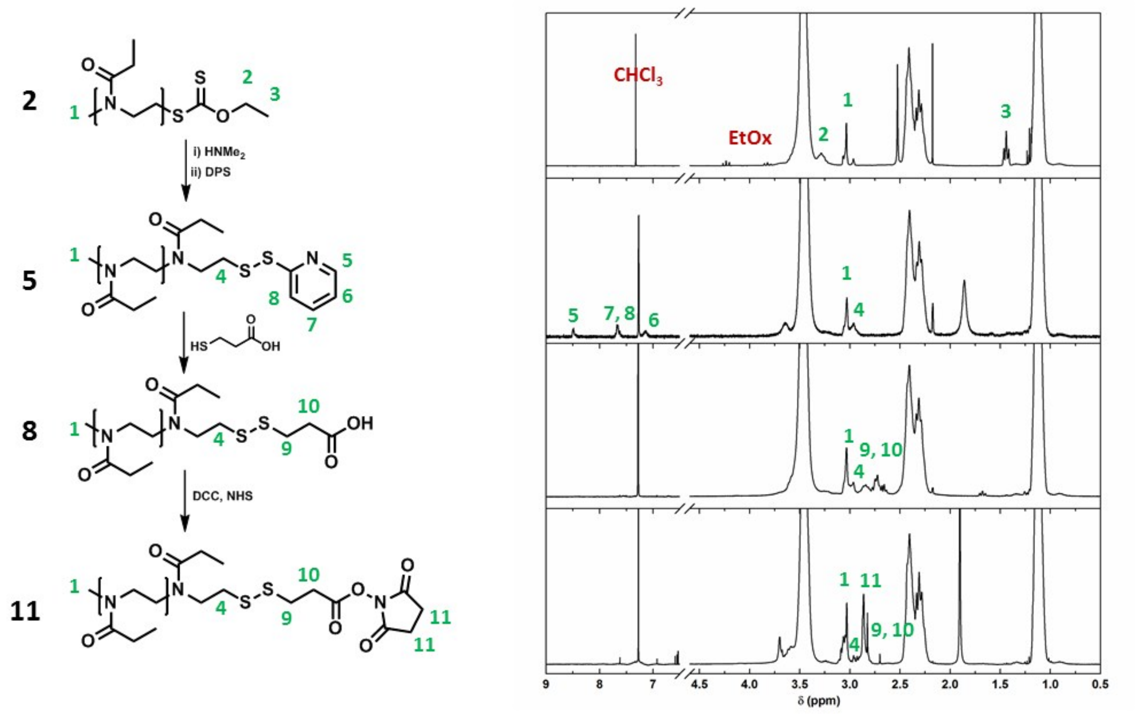

Figure S3: ^{1}H-NMR characterization of responsive polymers with DP = 20.

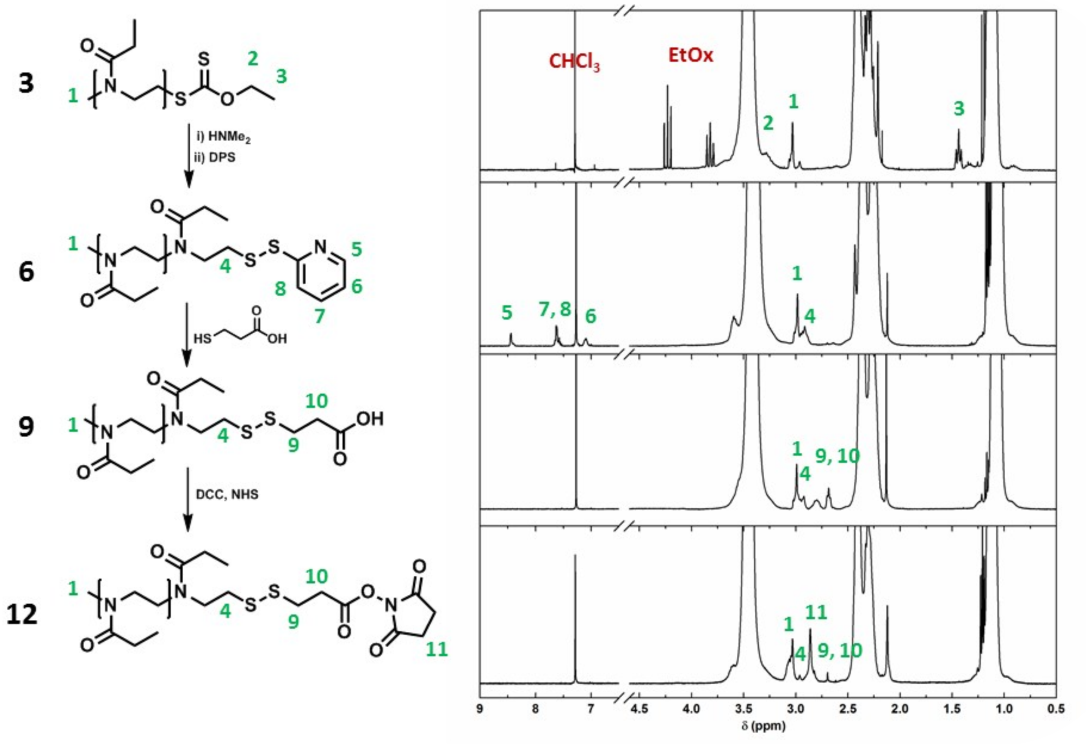

Figure S4: ^{1}H-NMR characterization of responsive polymers with DP = 45.

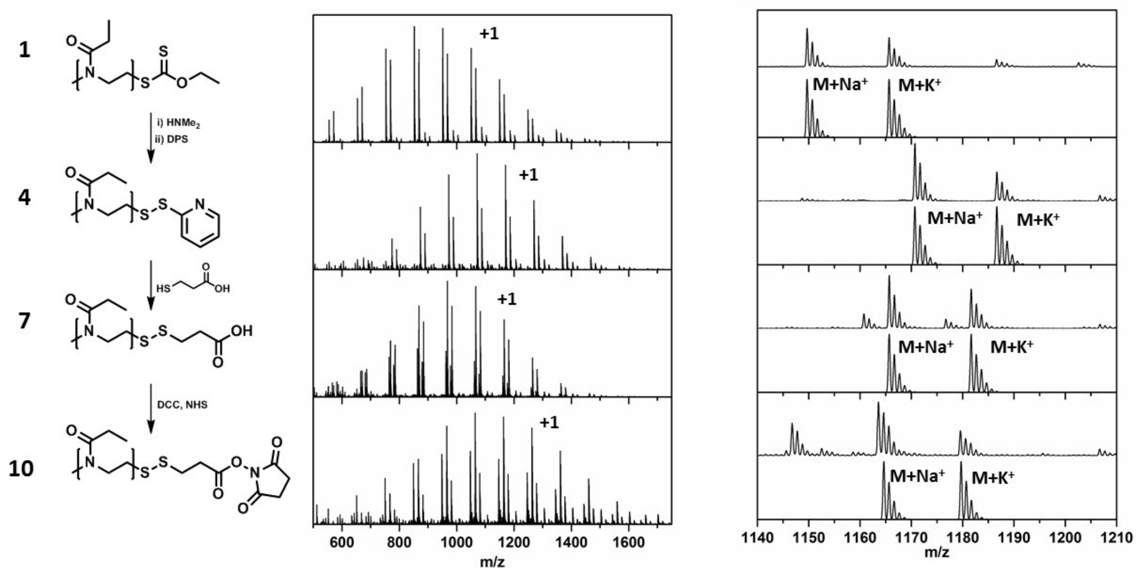

Figure S5: ESI-ToF-MS characterization of responsive polymers with a DP = 10.

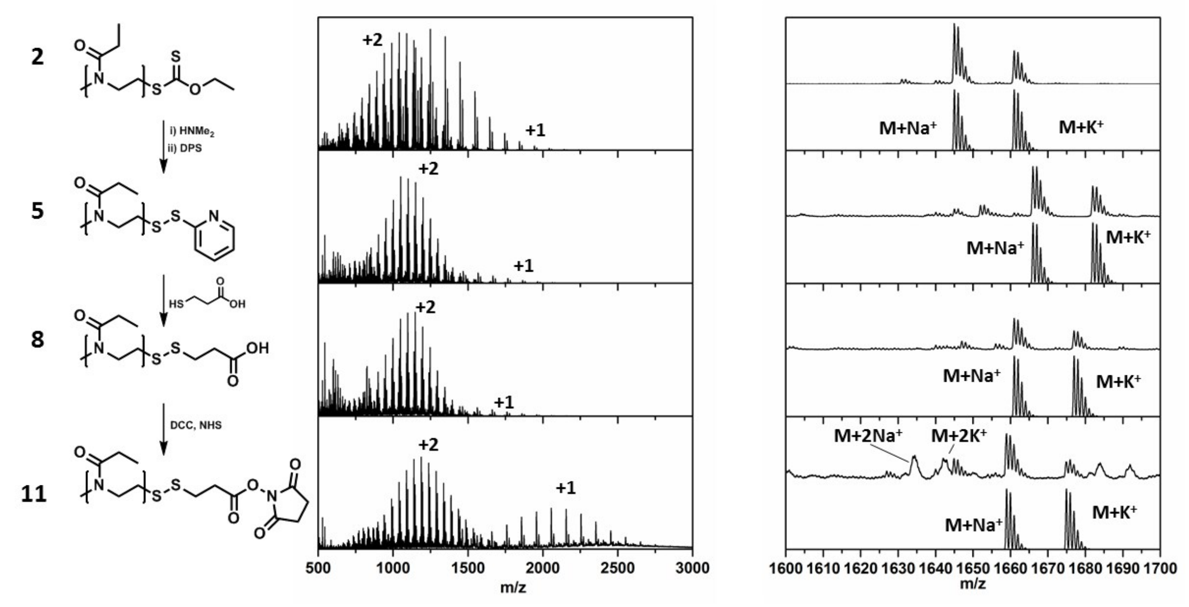

Figure S6: ESI-ToF-MS characterization of responsive polymers with a DP = 20.

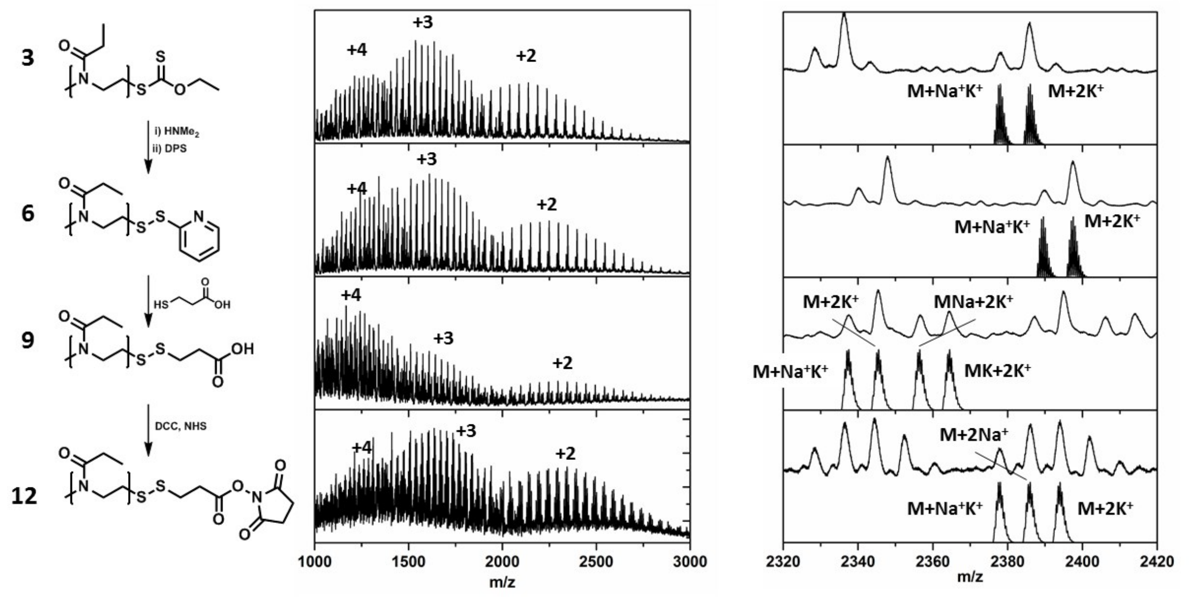

Figure S7: ESI-ToF-MS characterization of responsive polymers with a DP = 45.

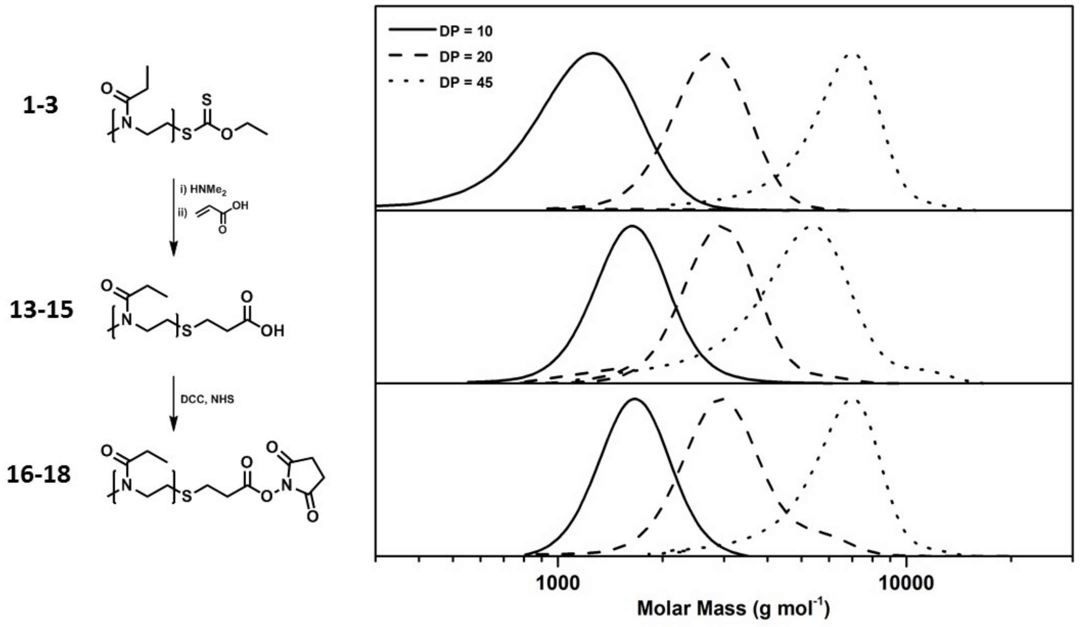

Figure S8: SEC characterization of non-responsive polymers with different DP values.

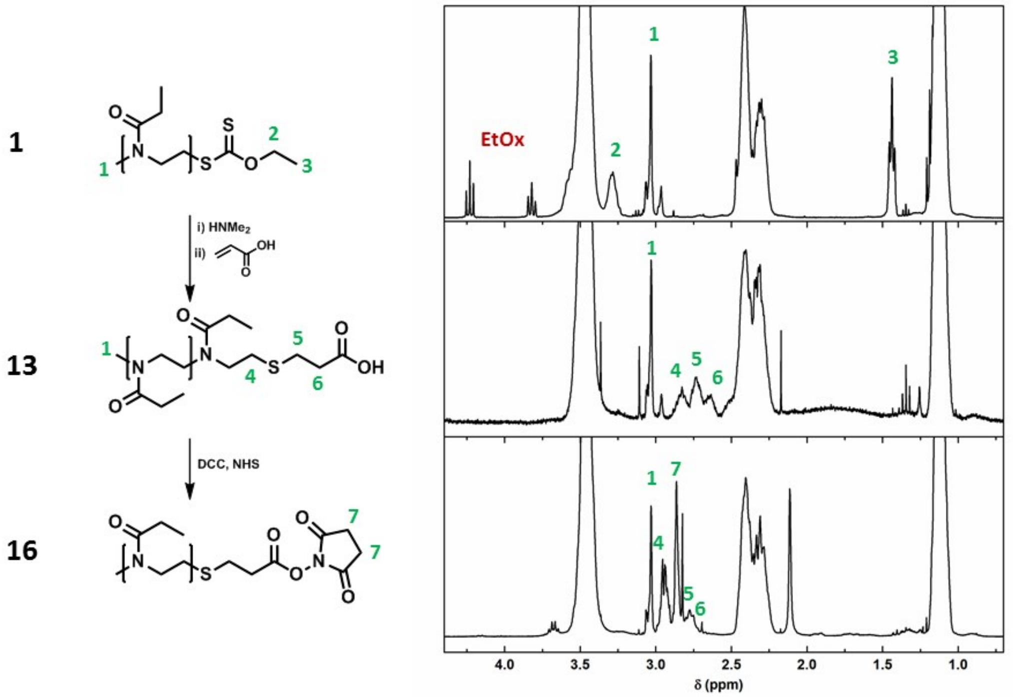

Figure S9: ^{1}H-NMR characterization of non-responsive polymers with DP = 10.

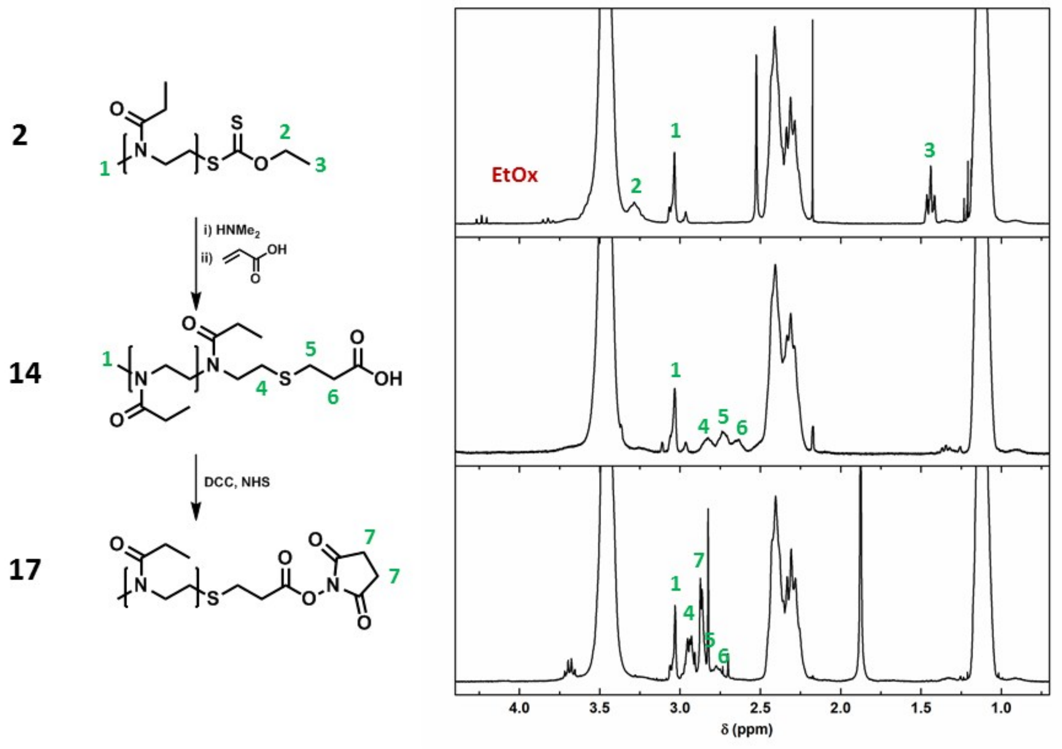

Figure S10: ^{1}H-NMR characterization of non-responsive polymers with DP = 20.

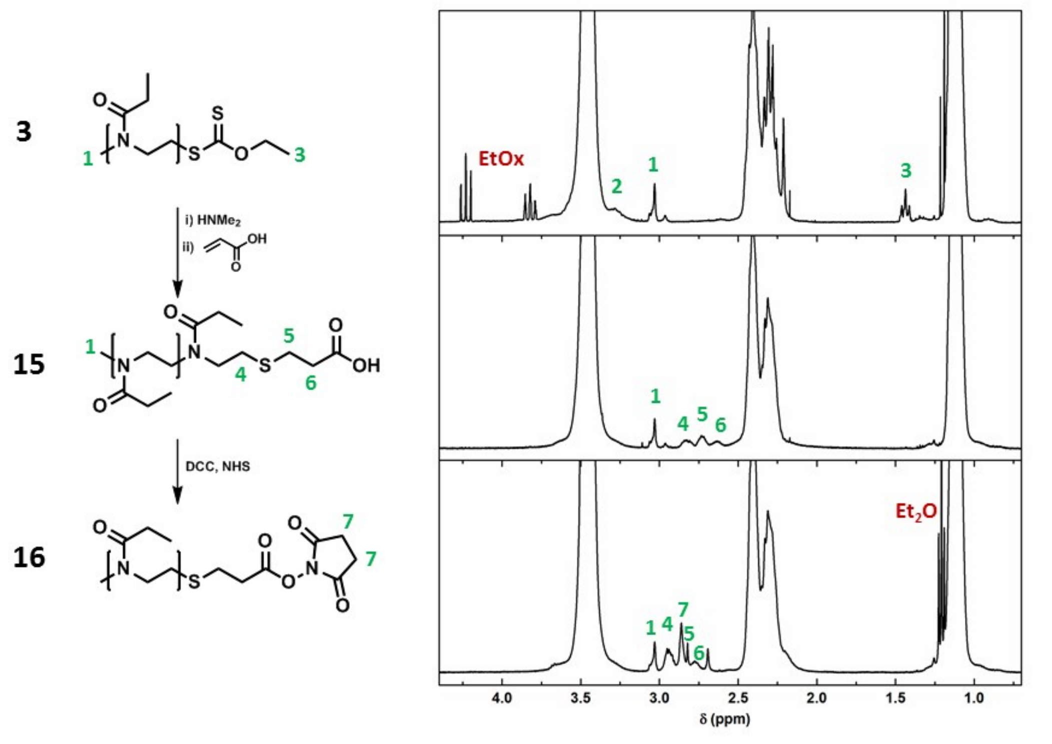

Figure S11: ^{1}H-NMR characterization of non-responsive polymers with DP = 45.

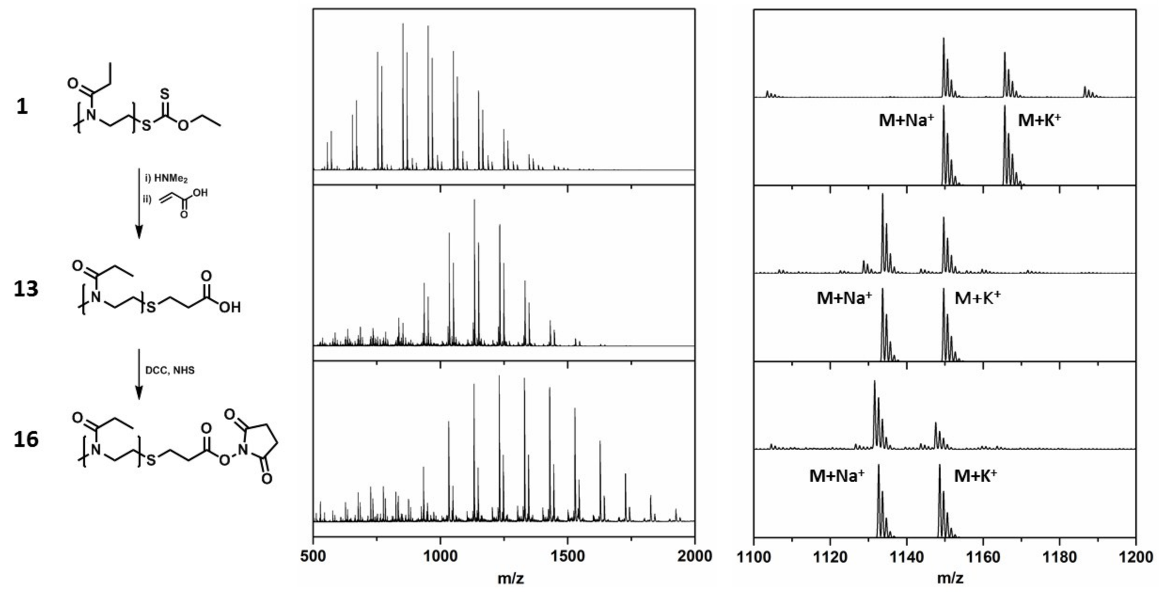

Figure S12: ESI-ToF-MS characterization of non-responsive polymers with a DP = 10.

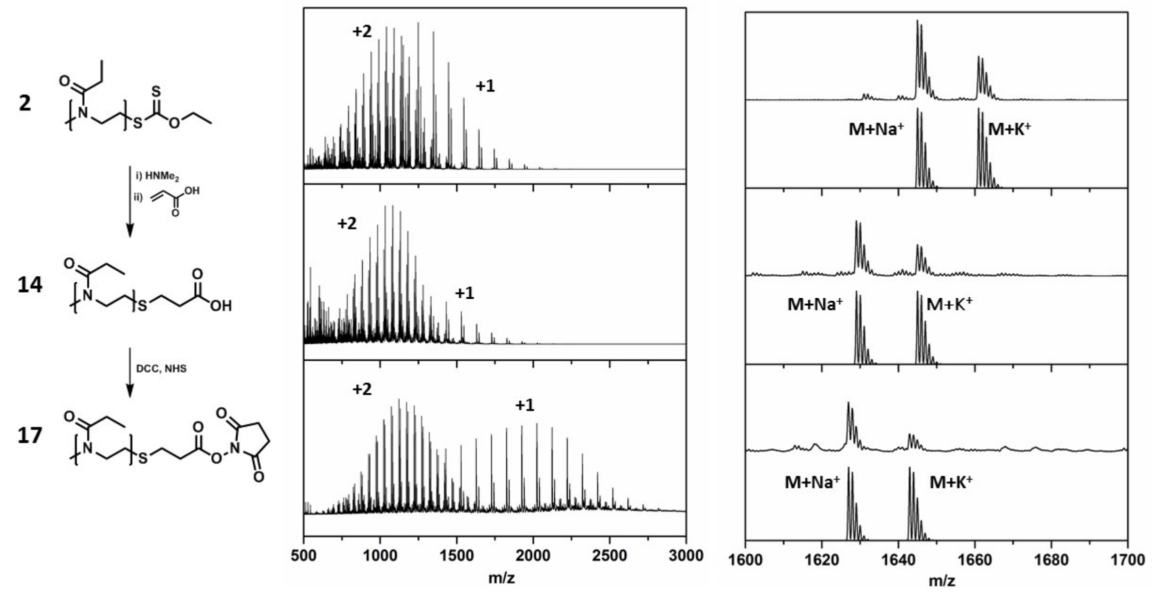

Figure S13: ESI-ToF-MS characterization of non-responsive polymers with a DP = 20.

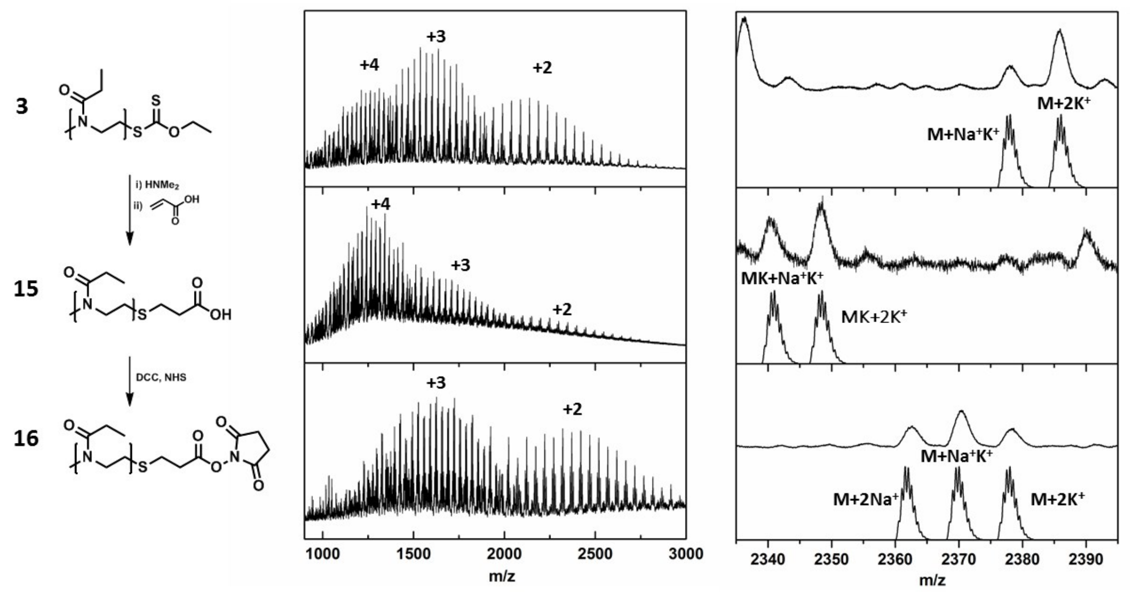

Figure S14: ESI-ToF-MS characterization of non-responsive polymers with a DP = 45.

Table S3: Characterization data of poly(2-ethyl-2-oxazoline)s with various functionalities.

Sample	End group	NMR DP	NMR M_n (g mol^{-1})	NMR DS	SEC M_n (g mol^{-1})	SEC Đ
**1**		8	900	0.98	1,000	1.20
**2**	Xanthate	18	1900	0.9	2,700	1.10
**3**		46	4700	0.9	5,900	1.10
**4**		10	1100	>0.9	1,000	1.20
**5**	S-S-Pyr	21	2200	>0.9	2,900	1.10
**6**		48	4900	0.88	6,100	1.11
**7**		10*	1100	n.d.	1,300	1.09
**8**	SS-COOH	21*	2200	>0.9	2,800	1.11
**9**		48*	4900	>0.9	6,200	1.11
**10**		10*	1200	>0.9	1,400	1.11
**11**	S-S-COONHS	21*	2300	>0.9	3,000	1.10
**12**		48*	5000	>0.9	6,400	1.14
**13**		10	1100	0.92	1,600	1.08
**14**	S-COOH	19	2000	0.95	2,800	1.08
**15**		46	4700	0.97	6,500	1.10
**16**		10*	1200	>0.9	1,600	1.06
**17**	S-COONHS	20*	2200	>0.9	2,900	1.12
**18**		46*	4800	>0.9	6,500	1.09

* DP could not be obtained from ^{1}H-NMR due to overlap of end group signals. Values were adopted from precursor polymers.

# Calculated from DP values.

‡ Measured in Chloroform/Triethylamine using a poly(styrene) calibration.

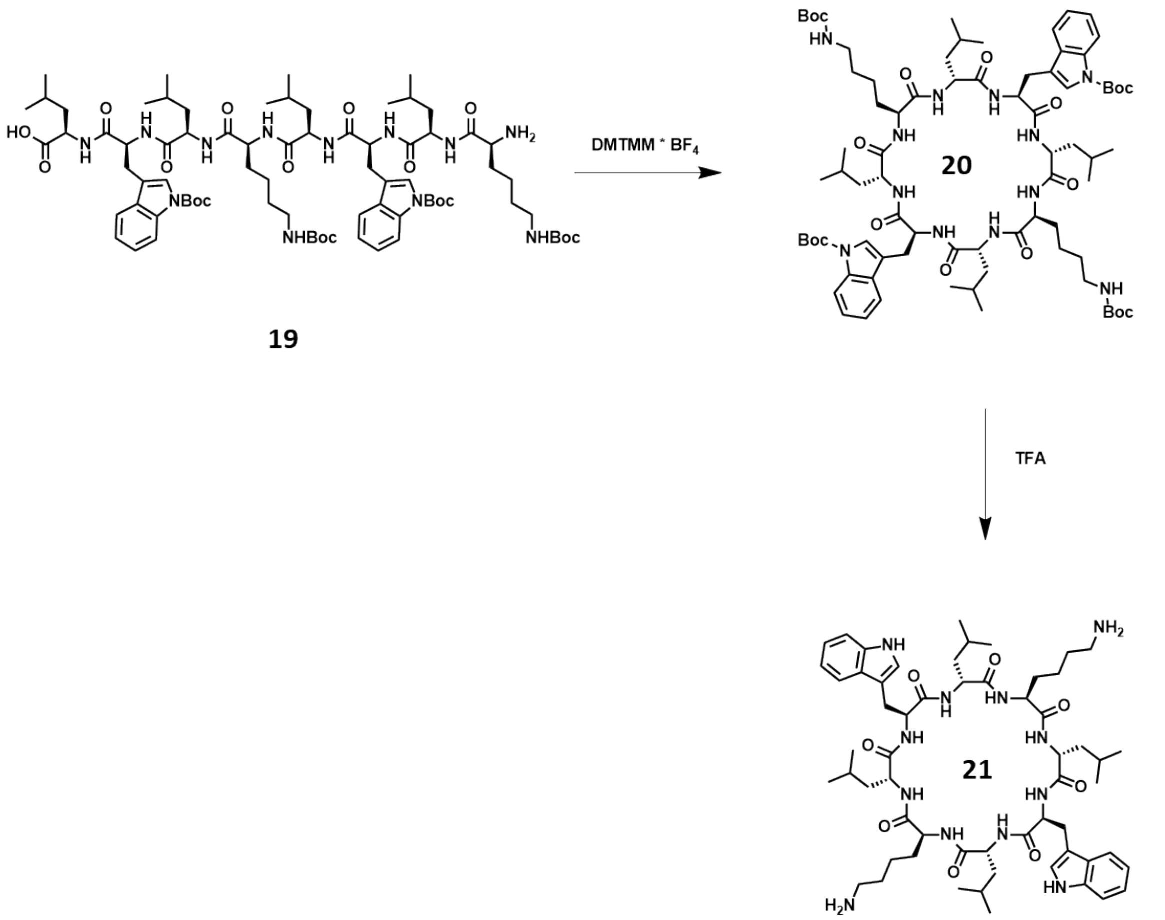

19
DMTMM * BF4
20
TFA
21

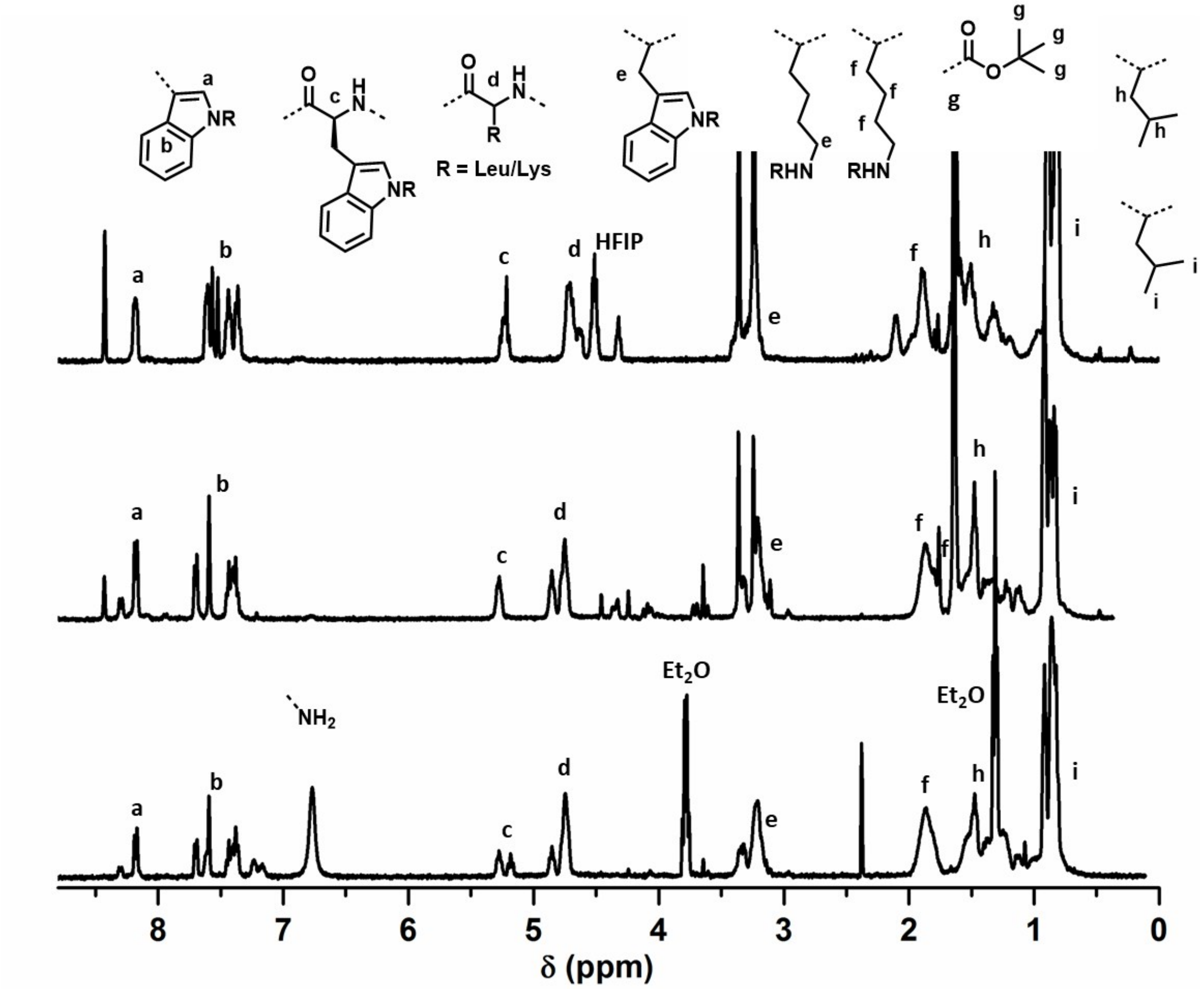

R = Leu/Lys
RHN
HFIP
Et2O
NH2
δ (ppm)

Figure S15: ^{1}H-NMR spectroscopy of linear (**19**), cyclized (**20**), as well as deprotected cyclic peptide (**21**). Spectra were recorded in deuterated trifluoro acetic acid (TFA(d)).

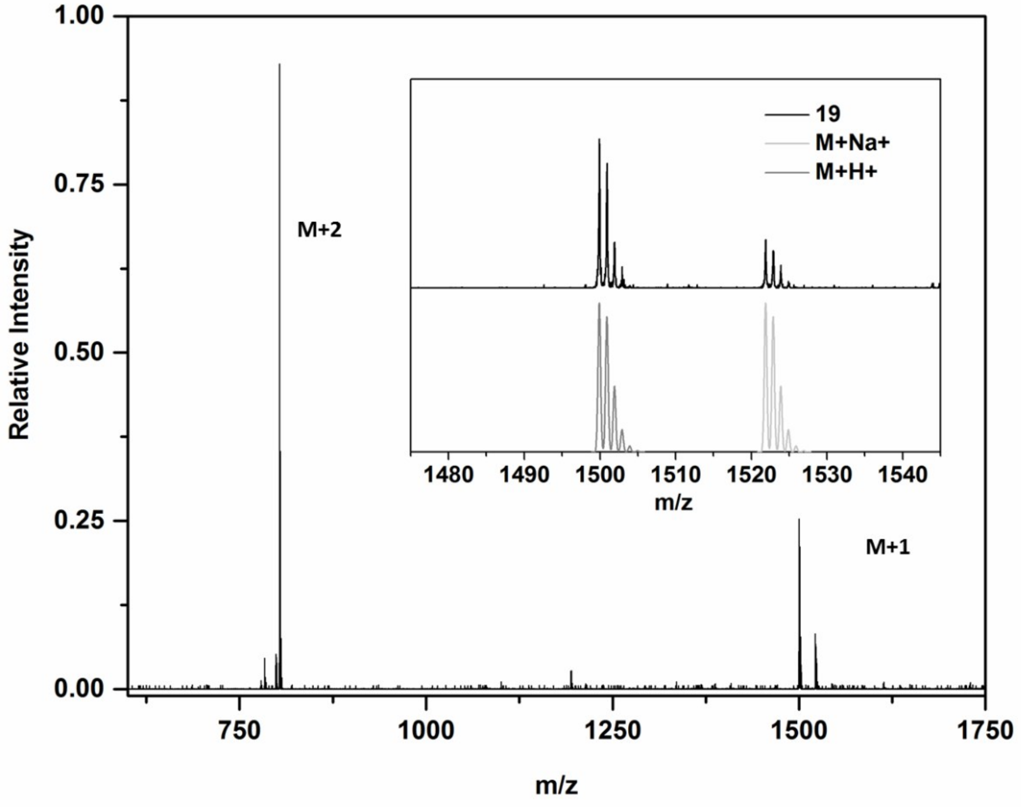

Figure S16: ESI-ToF-MS of linear peptide (**19**); Measured: 1499.902 m/z (M+Na^{+}), simulated: 1499.892 m/z;

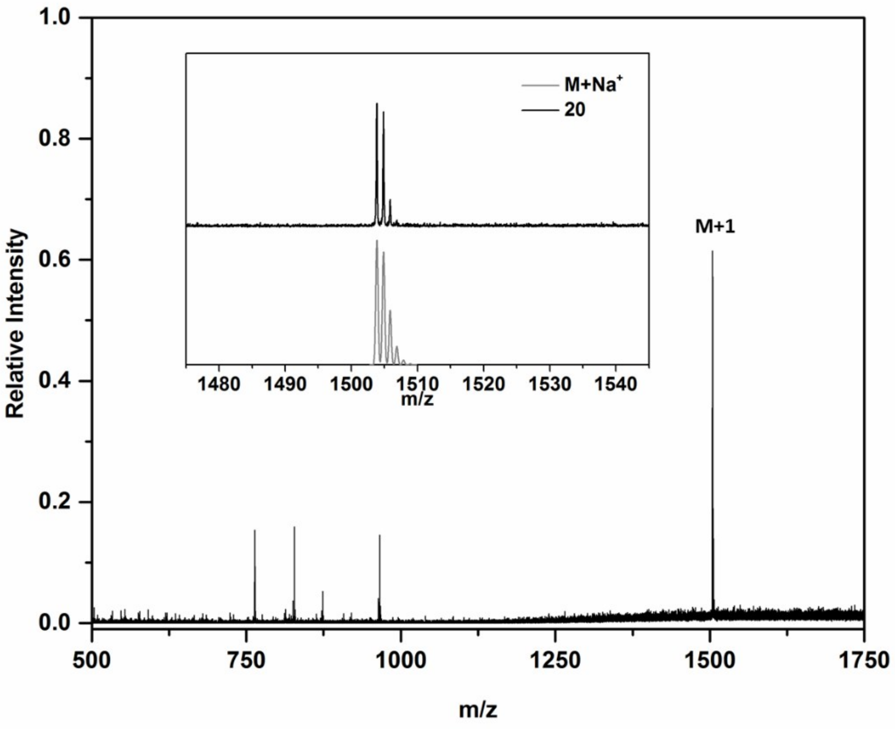

Figure S17: ESI-ToF-MS of cyclic protected peptide (**20**); Measured: 1503.892 m/z ($M+H^{+}$), simulated: 1503.884 m/z;

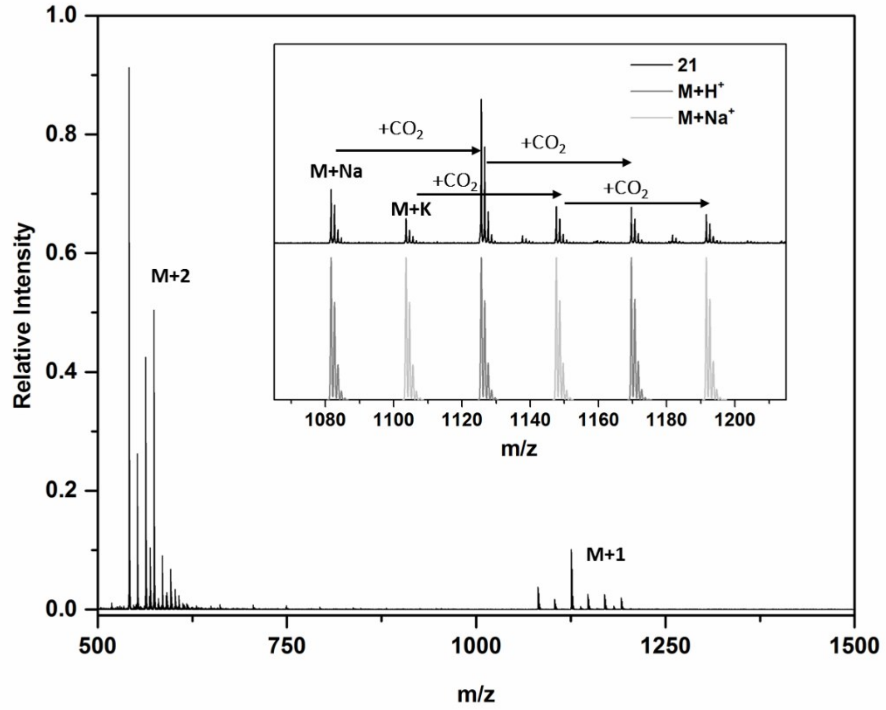

Figure S18: ESI-ToF-MS of cyclic deprotected peptide (**21**); Measured: 1081.690 m/z ($M+H^+$), simulated: 1081.692 m/z;

The additional peaks representing CO_2 adducts of the desired substance are a product of residual fragments of the Boc protection group. As this does not inflict any problems for later synthetic steps, it was not removed (as possible by e.g. vacuum exposure).

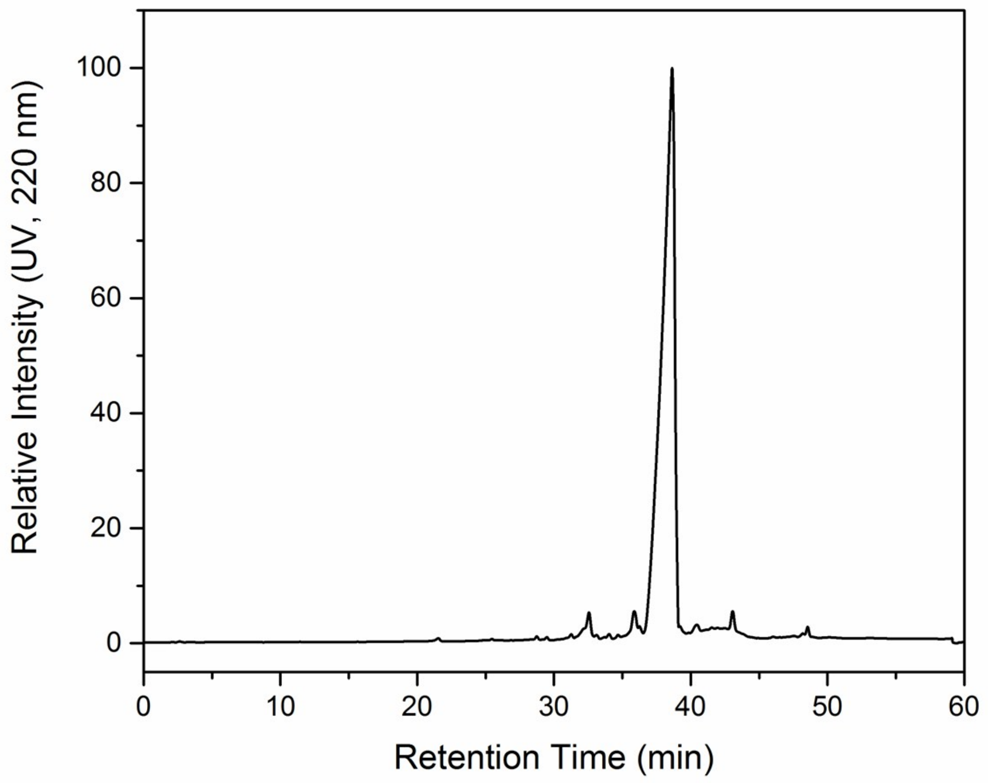

Figure S19: HPLC of linear peptide (**19**).

Note: Cyclic protected peptide (**20**) could not be analysed by HPLC as the molecule was not soluble in suitable solvents.

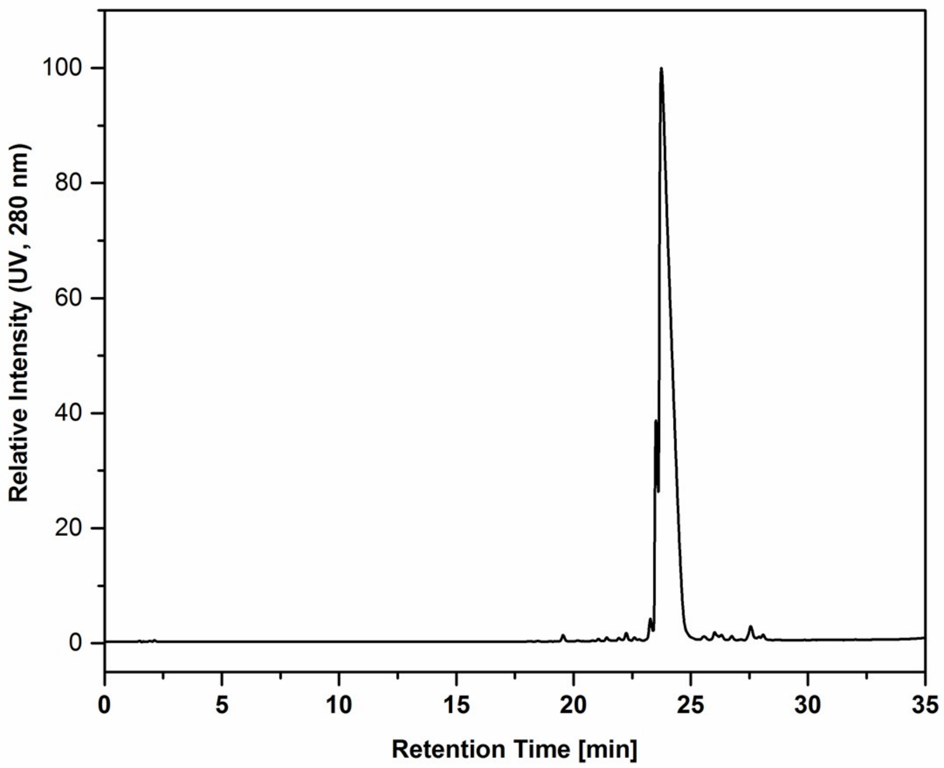

Figure S20: HPLC of cyclic deprotected peptide (**21**)

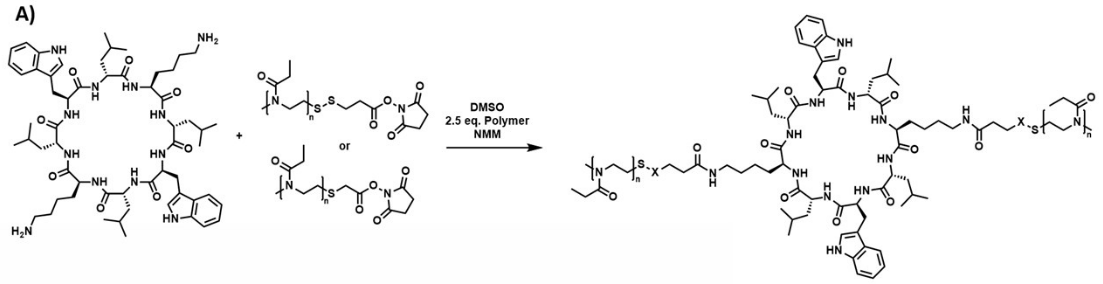

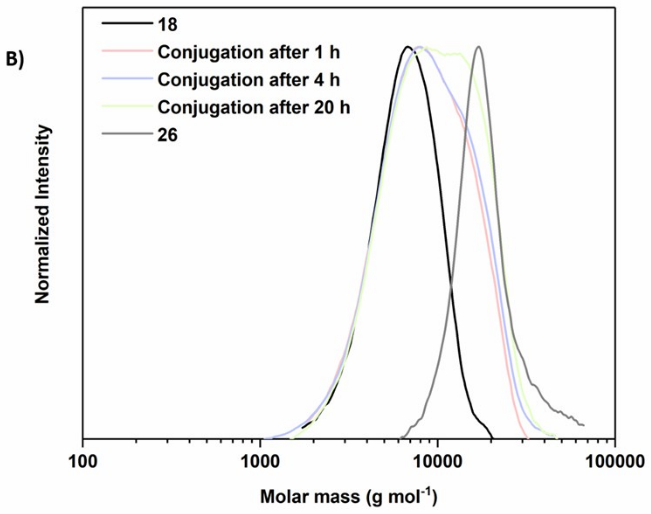

Sample	Connection	n	Yield (%)
22	CH_2-S-S-CH_2	10	53
23	CH_2-S-CH_2	10	51
24	CH_2-S-S-CH_2	20	60
25	CH_2-S-CH_2	20	82
26	CH_2-S-S-CH_2	45	46
27	CH_2-S-CH_2	45	38

Scheme S2: A) Schematic representation of the conjugation of PEtOx with varying DP and linkage to cyclic peptides; B) SEC-traces of the conjugation of polymer 18 to cyclic peptide 21 as a function of conjugation time and after purification.

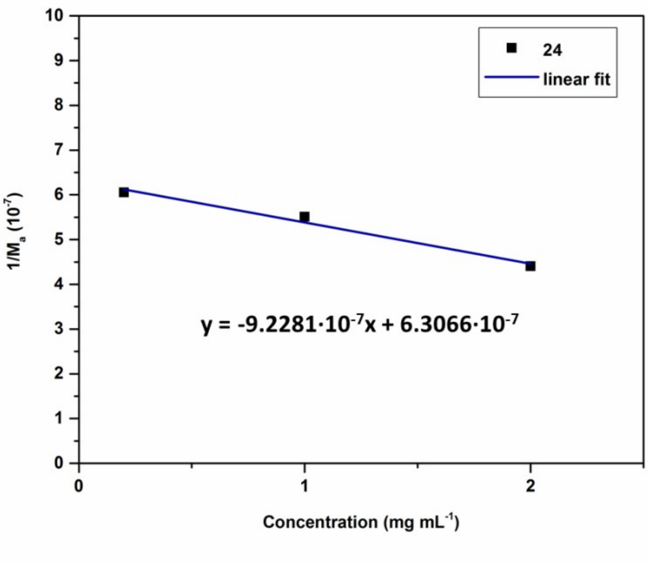

Figure S21: SLS data of conjugate **24** measured in water.

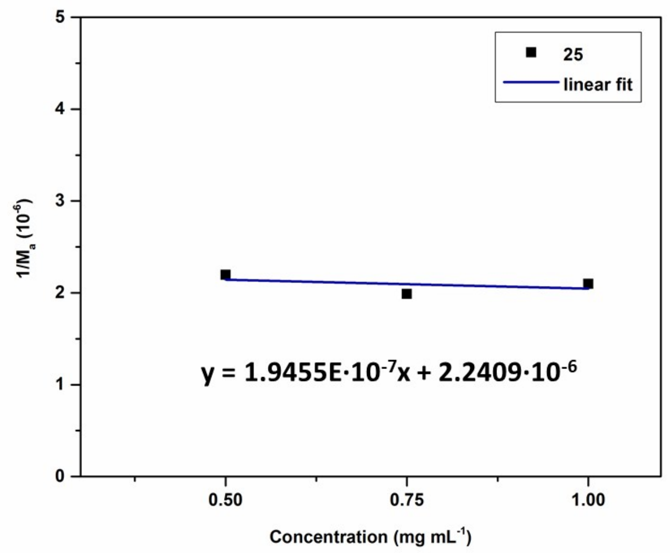

Figure S22: SLS data of conjugate **25** measured in water

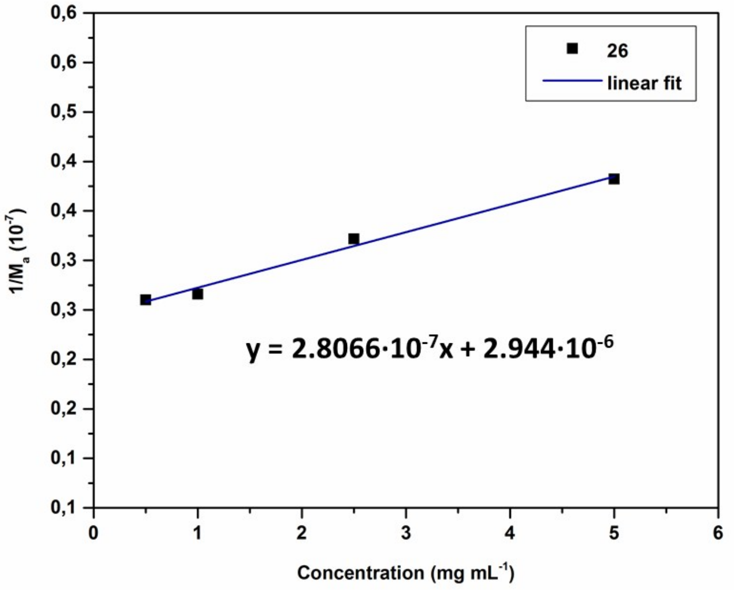

Figure S23: SLS data of conjugate **26** measured in water.

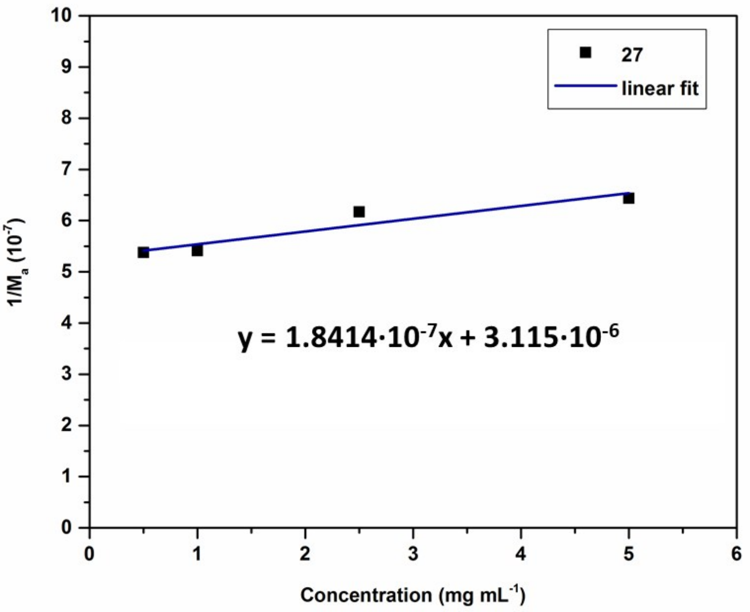

Figure S24: SLS data of conjugate **27** measured in water.

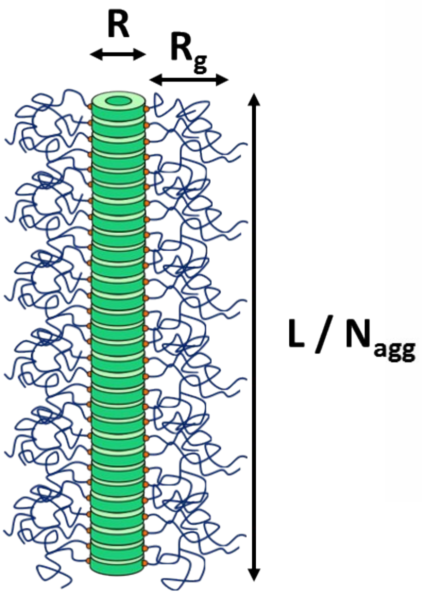

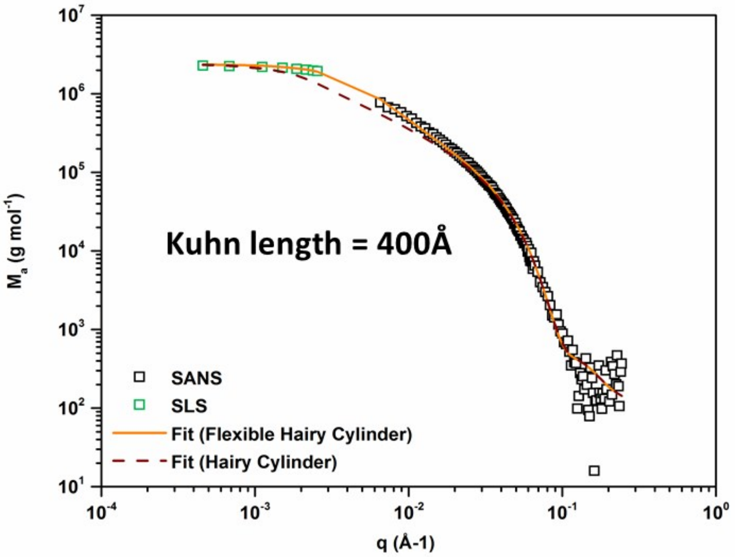

	Fit	SLS
L =	1900 Å	
N_{agg} =	400	278
R =	6.4 Å	
R_g =	16.5 Å	

Figure S25: SANS an SLS data of conjugate **24** fitted with hairy cylinder and flexible hairy cylinder model.

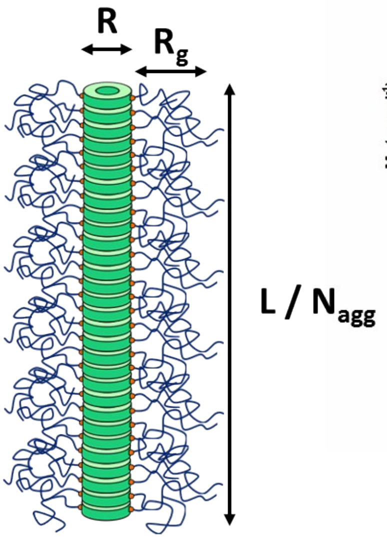

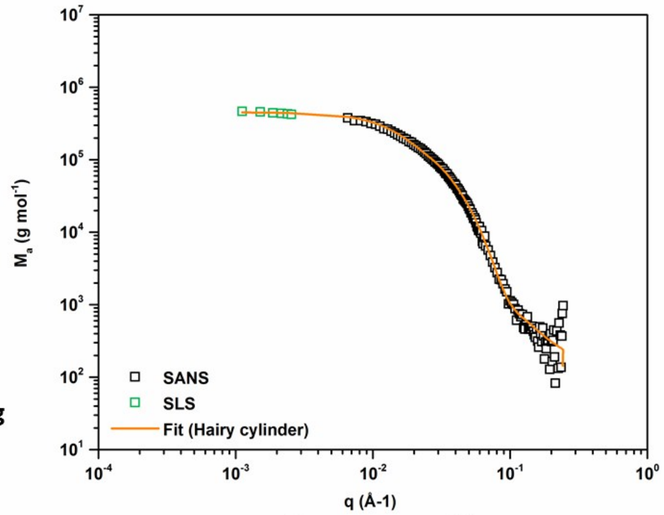

	Fit	SLS
L =	270 Å	
N_{agg} =	58	90
R =	6.4 Å	
R_g =	17.8 Å	

Figure S26: SANS an SLS data of conjugate **25** fitted with hairy cylinder model.

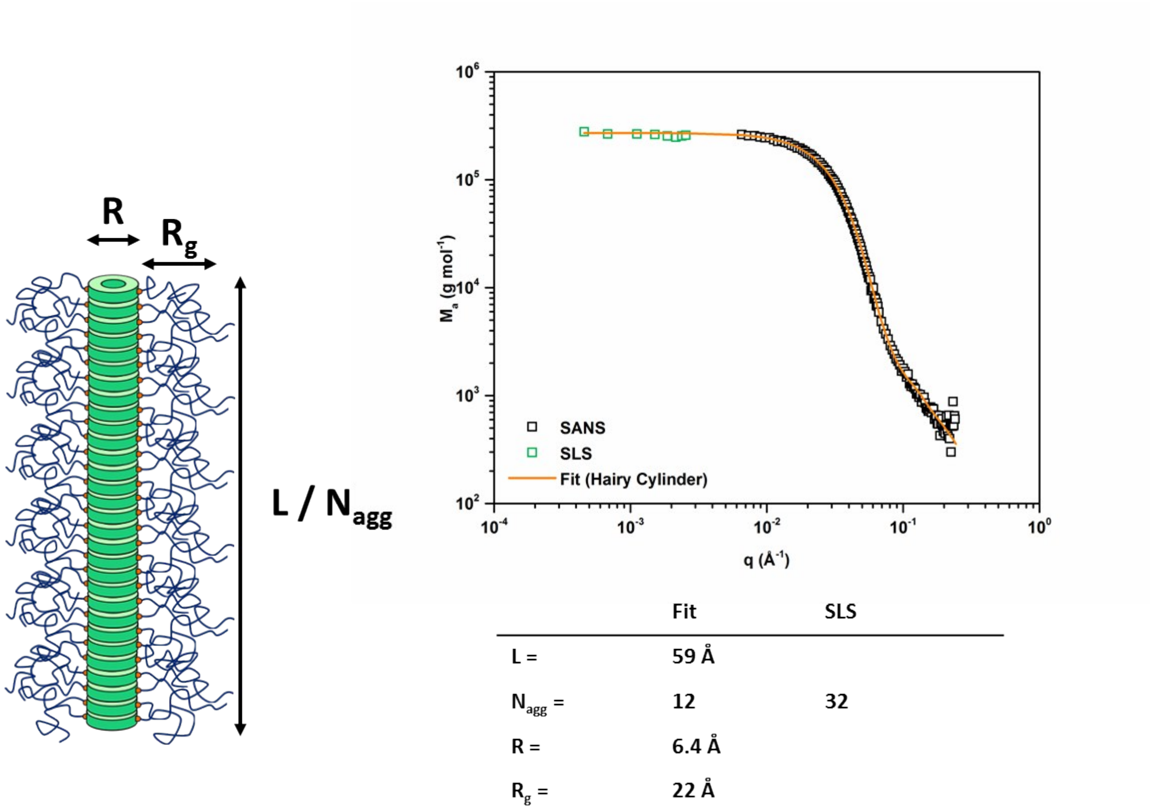

	Fit	SLS
L =	59 Å	
N_{agg} =	12	32
R =	6.4 Å	
R_g =	22 Å	

Figure S27: SANS an SLS data of conjugate **26** fitted with hairy cylinder model.

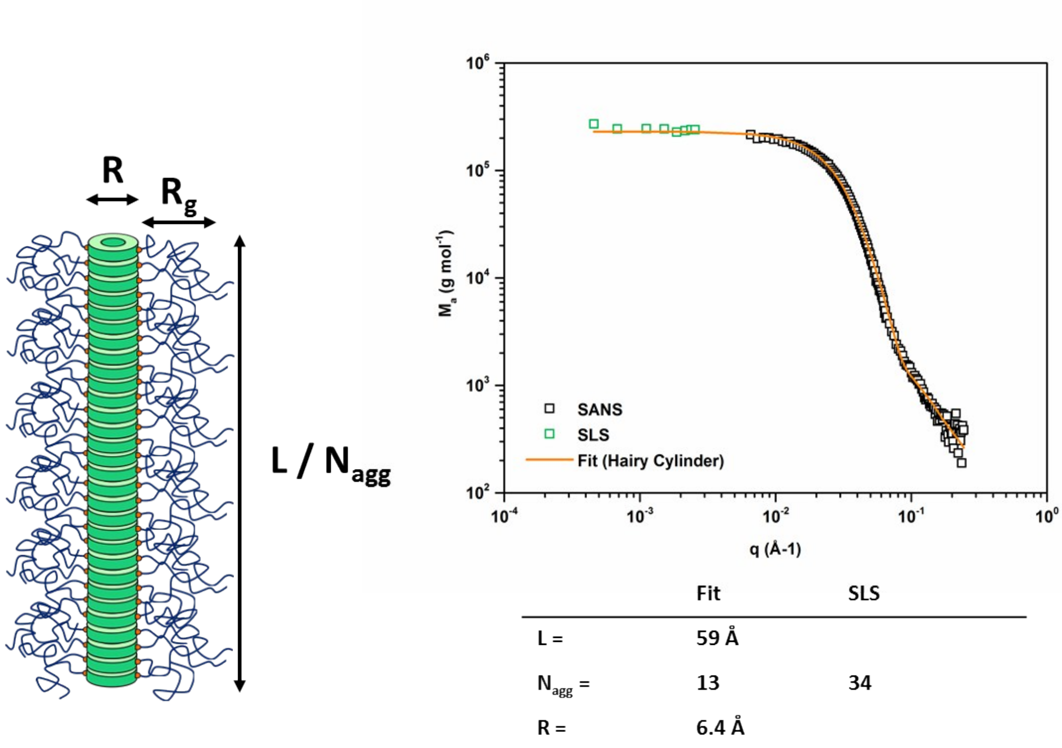

	Fit	SLS
L =	59 Å	
N_{agg} =	13	34
R =	6.4 Å	
R_g =	22 Å	

Figure S28: SANS an SLS data of conjugate **27** fitted with hairy cylinder model.

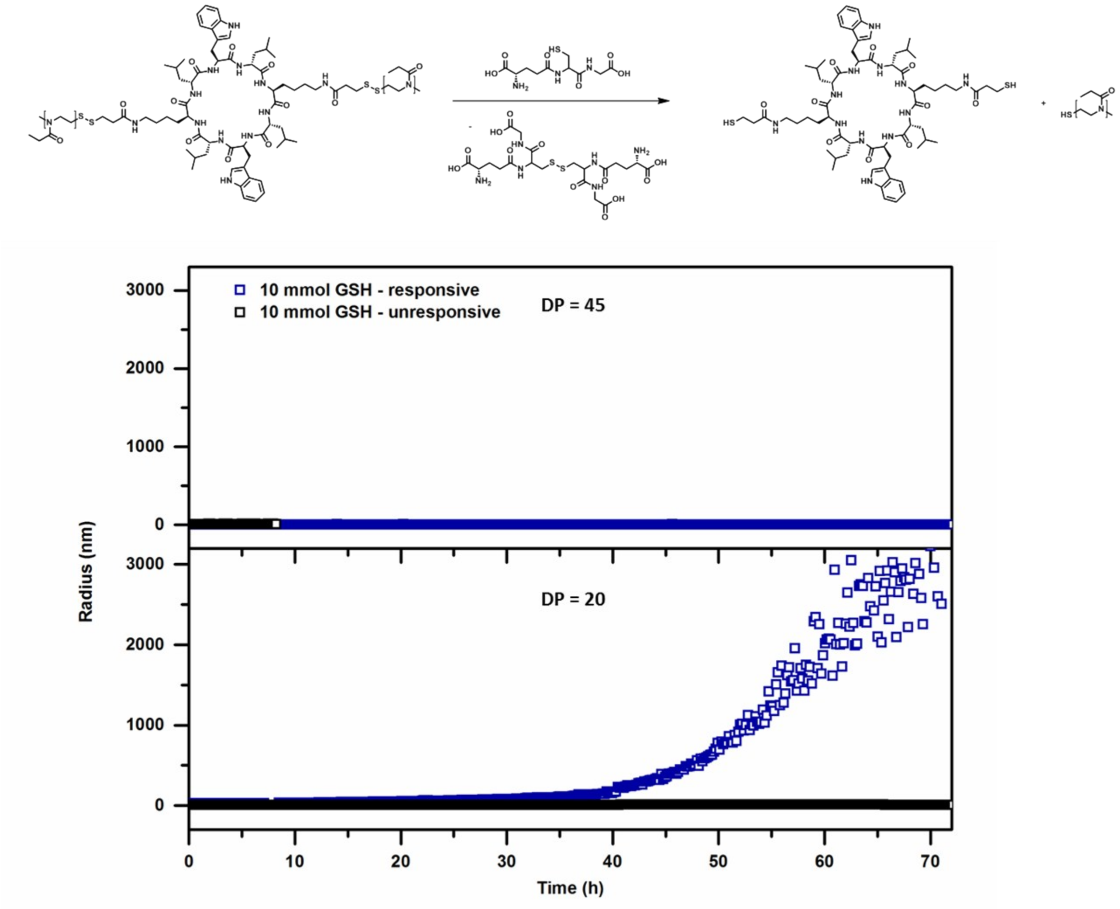

Figure S29: Glutathione (GSH) mediated detachment of polymer arms from conjugates **24** (DP = 20) and **26** (DP = 45) at a concentration of 1 mg mL^{-1} and a total GSH concentration of 10 mM in water monitored by DLS.

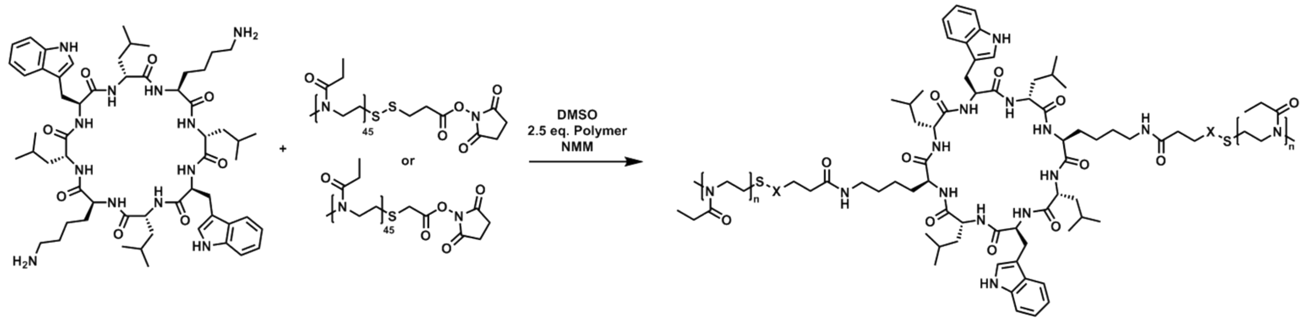

Sample	Responsive Polymer (%)	Non-responsive polymer (%)
**28**	25	75
**29**	50	50
**30**	75	25

Scheme S3: Synthesis and composition of polymer-CPNT conjugates with a mixed polymer shell.

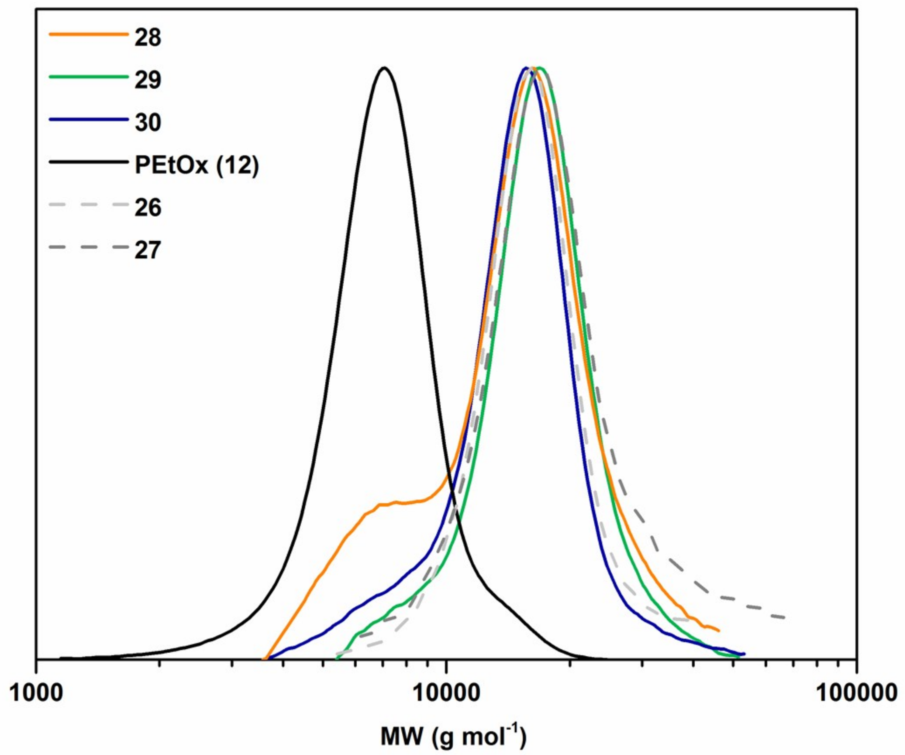

Figure S30: SEC characterization of conjugates with mixed polymer shell (**28 – 30**) compared to precursor polymer (**12**) and responsive (**26**) as well as non-responsive (**27**)

conjugates. The low molecular weight shoulder of Compound **28** corresponds to a small population of peptide with one polymer arm. Measurements were done in DMF (LiBr) and PS calibration was used.

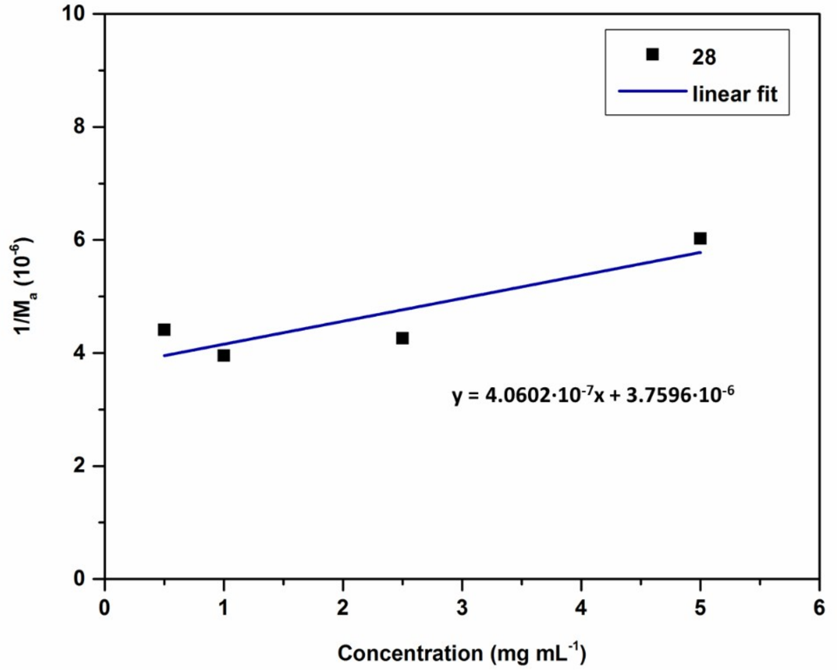

Figure S31: SLS data of conjugate **28** measured in water prior to arm detachment

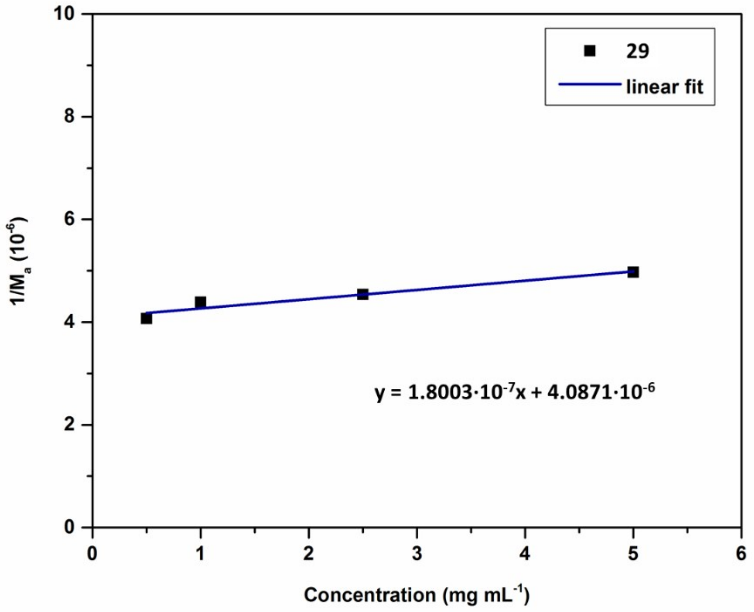

Figure S32: SLS data of conjugate **29** measured in water prior to arm detachment.

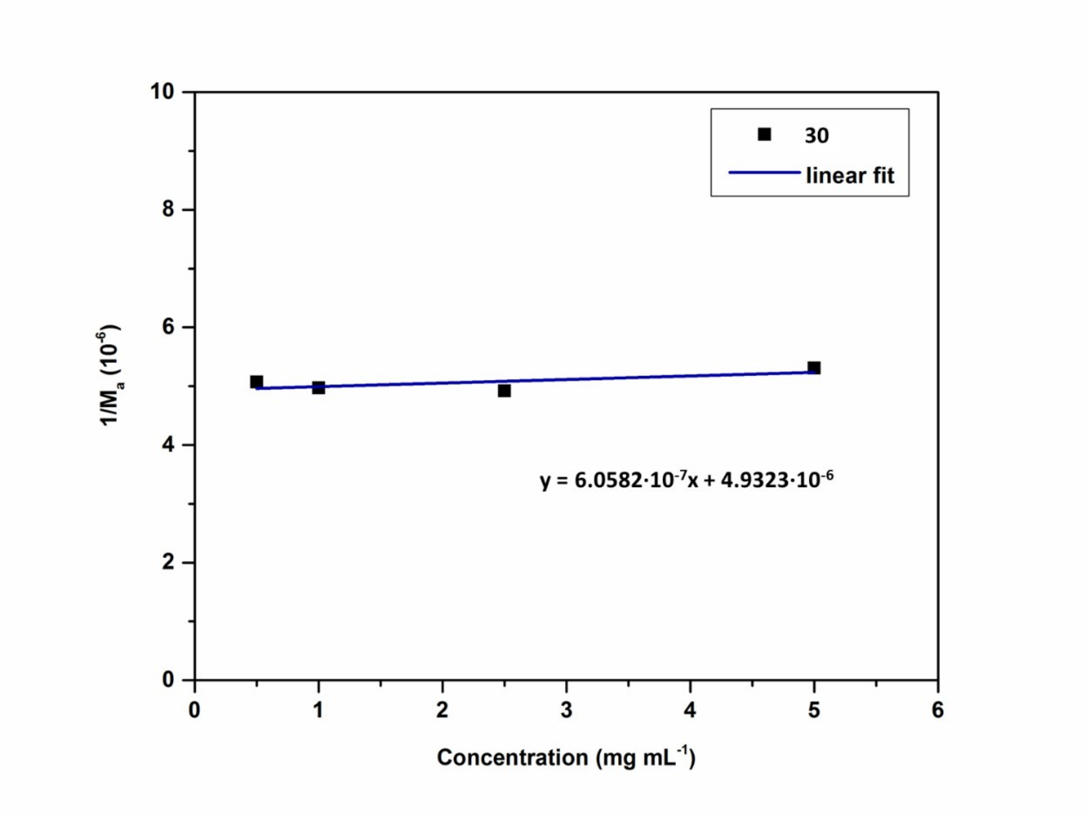

Figure S33: SLS data of conjugate **30** measured in water prior to arm detachment.

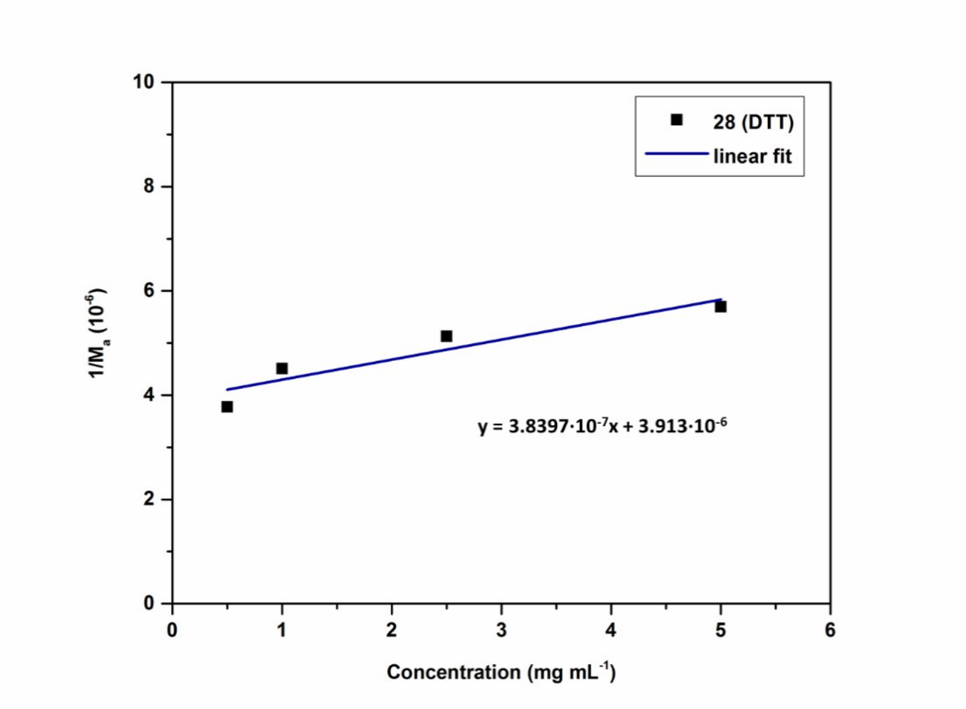

Figure S34: SLS data of conjugate **28** measured in water after detachment of responsive polymer arms using DTT.

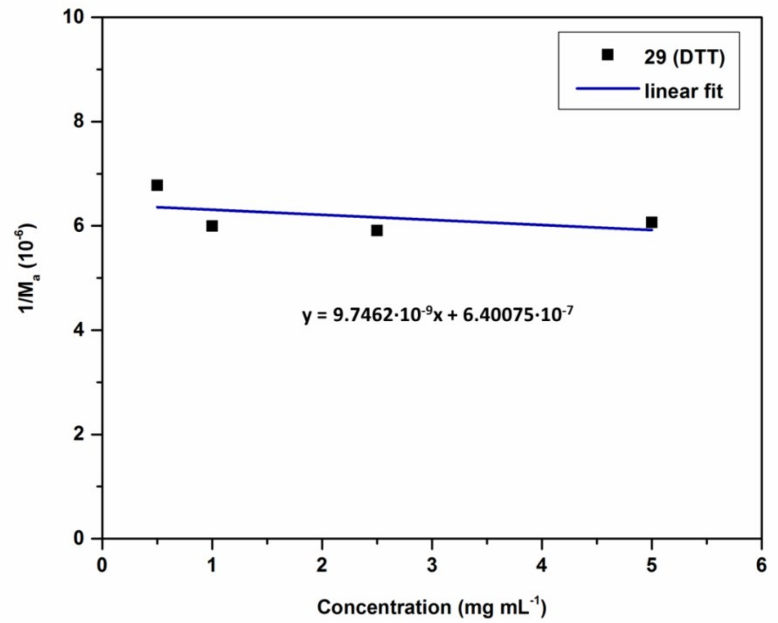

Figure S35: SLS data of conjugate **29** measured in water after detachment of responsive polymer arms using DTT.

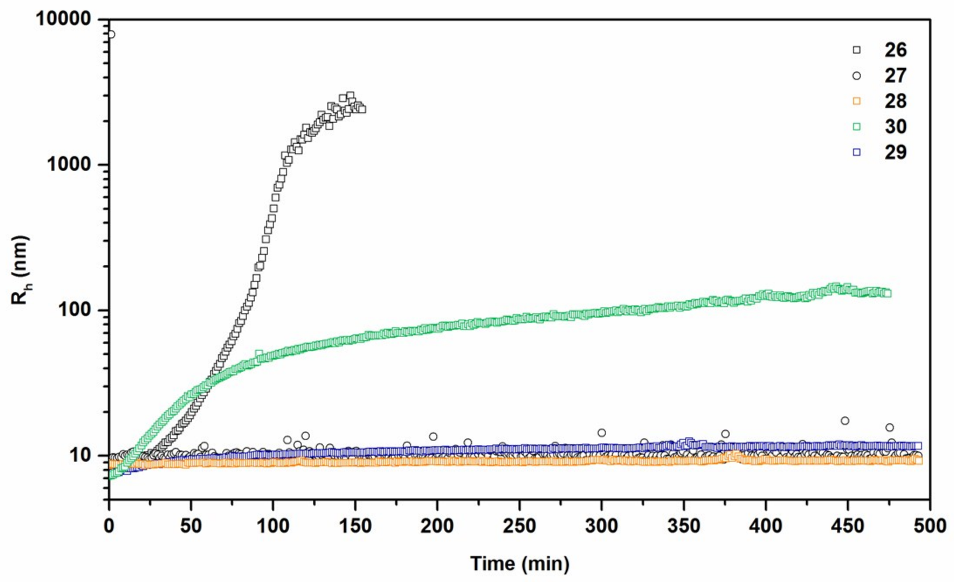

Figure S36: DTT (30 mM) mediated detachment of polymer arms from conjugates (1 mg mL^{-1})with a mixed polymer shell (**28 – 30**) compared to responsive (**26**) and non-responsive (**27**) peptide monitored by DLS in water.

Table S4: Aggregation behaviour of nanotubes with a mixed polymeric shell (responsive and non-responsive) before and after treatment with reduction by DTT.

Sample	Responsive Polymer (%)	Non-responsive. Polymer (%)	SLS Prior Reduction		SLS After Reduction	
			Mw (SLS)	N_{agg}	Mw (SLS)	N_{agg}
**28**	25	75	267,000	26	256,000	32
**29**	50	50	244,000	24	1,620,000	279
**30**	75	25	203,000	20	-*	-*

* - Scattering data could not be obtained due to precipitation.

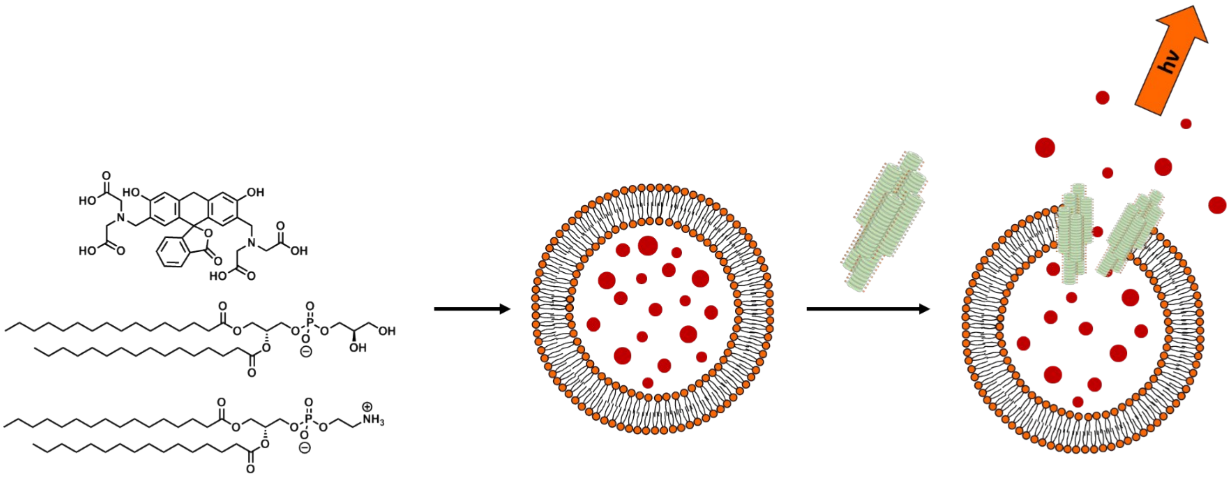

Scheme S4: Schematic representation of liposome synthesis based on bacterial phospholipids (phosphatidylethanolamine and phosphatidylglycerol mimicking E. coli) and dye leakage induced by the presence of cyclic peptide nanotubes.

References

1. Brown, W., *Light Scattering: Principles and Development*. Clarendon Press: 1996.
2. Zemb Th., L. P., *Neutron, X-rays and Light. Scattering Methods Applied to Soft Condensed Matter* Th. Zemb Editor: North Holland, 2002; p 552.

3. Bressler, I.; Kohlbrecher, J.; Thunemann, A. F., SASfit: a tool for small-angle scattering data analysis using a library of analytical expressions. *J. Appl. Crystallogr.* **2015,** *48* (5), 1587-1598.
4. Pedersen, J., Form factors of block copolymer micelles with spherical, ellipsoidal and cylindrical cores. *Journal of Applied Crystallography* **2000,** *33* (3 Part 1), 637-640.
5. Lienkamp, K.; Kumar, K.-N.; Som, A.; Nüsslein, K.; Tew, G. N., "Doubly Selective" Antimicrobial Polymers: How Do They Differentiate between Bacteria? *Chemistry – A European Journal* **2009,** *15* (43), 11710-11714.
6. Vichai, V.; Kirtikara, K., Sulforhodamine B colorimetric assay for cytotoxicity screening. *Nature Protocols* **2006,** *1*, 1112.
